# Microgeographic genomic variation and connectivity in an endangered semiaquatic mammal

**DOI:** 10.1186/s12862-025-02460-1

**Published:** 2025-10-23

**Authors:** Sara Sampaio, Soraia Barbosa, José A. Andrés, Lorenzo Quaglietta, Steven Bogdanowicz, Paulo Barros, Michaela Horníková, Joana Paupério, Paulo C. Alves, Jeremy B. Searle

**Affiliations:** 1 CIBIO/InBIO, Research Center in Biodiversity and Genetic Resources, University of Porto, Vairão, Portugal; 2 Department of Biology, Faculty of Sciences of University of Porto, Porto, Portugal; 3 BIOPOLIS Program in Genomics, Biodiversity and Land Planning, CIBIO, Campus de Vairão, Vairão, Portugal; 4 Department of Ecology and Evolutionary Biology, Cornell University, Ithaca, NY USA; 5 CIBIO/InBIO, Research Center in Biodiversity and Genetic Resources, Institute of Agronomy, University of Lisbon, Lisbon, Portugal; 6 AEPGA – Associação para o Estudo e Protecção do Gado Asinino – Largo da Igreja, Atenor (Vimioso), Portugal; 7 Fluvial and Terrestrial Ecology Laboratory, University of Trás-os-Montes and Alto Douro, Vila Real, Portugal; 8 Laboratory of Molecular Ecology, Institute of Animal Physiology and Genetics, Czech Academy of Sciences, Liběchov, Czechia; 9 Present Address: EMBL-EBI, European Molecular Biology Laboratory, European Bioinformatics Institute, Wellcome Genome Campus, Hinxton, Cambridge, UK; 10 EBM, Biological Station of Mértola, Mértola, Portugal

**Keywords:** Population genomics, *Galemys pyrenaicus*, Connectivity, Conservation genomics

## Abstract

The Iberian desman (*Galemys pyrenaicus*), a semiaquatic mammal endemic to southwestern Europe, is listed as Endangered by the IUCN due to substantial range and population decline in recent years, with its populations now largely limited to river headwaters. This restriction to upstream locations highlights the importance of documenting genetic variation and population connectivity in Iberian desmans for effective conservation strategies. While previous studies have revealed phylogeographic structure across the range of the species, gaps remain in our understanding of the microgeographic dynamics that shape genetic exchange within specific geographic regions. This study first combined our newly generated SNP data with previous datasets to further explore genetic structure across the entire Iberian desman distribution using the 115 individuals now available. Due to differences in methodology between our study and previous studies, only 110 SNPs were available for comparison across all individuals. Nevertheless, the analysis with these SNPs confirmed the presence of the five major phylogeographic units previously described and validated the assignment of our newly generated dataset to the Occidental phylogeographic unit. Focusing on our newly generated data, we explored the microgeographic dynamics of this Occidental phylogeographic unit with a higher-resolution genomic dataset (7,604 SNPs, 14 individuals). This analysis provided evidence of isolation-by-distance (IBD), suggesting that gene flow decreases with increasing geographic distance and that dispersal occurs primarily over short distances. Focussing on the Douro river system, our genomic clustering results showed both connectivity along the best-sampled river (Sabor) and between headwaters of this and headwaters from a closely located river (Tua). Our IBD results were consistent with this, indicating riverine dispersal as well as a combination of riverine and overland dispersal at headwaters. These results highlight the importance of both aquatic and terrestrial corridors in upstream areas for maintaining connectivity. Thus, conservation efforts should prioritize the protection and restoration of riparian and terrestrial corridors, particularly in fragmented landscapes, to mitigate isolation and preserve genetic diversity in the Iberian desman. This study underscores the value of genomic approaches in conservation and contributes to a deeper understanding of the ecological and evolutionary processes that maintain population connectivity in an endangered species.

**Supplementary Information:**

The online version contains supplementary material available at 10.1186/s12862-025-02460-1.

## Background

Understanding microgeographic population structure is essential for conservation biology, particularly in species inhabiting fragmented or isolated habitats, because of what it reveals about population connectivity and local adaptive potential, including resilience to current environmental change (e.g [1–3]). In freshwater ecosystems, dams and other barriers, habitat degradation, and climate change can lead to genetic isolation and can increase the risk of inbreeding in aquatic species by reducing gene flow and connectivity [4–6]. Similar processes can also affect semiaquatic mammals, thereby restricting populations to headwater streams [7, 8]. However, because semiaquatic species depend on both terrestrial and aquatic habitats, overland dispersal among proximate headwaters can potentially promote gene flow between populations [9]. While semiaquatic amphibians and reptiles have been relatively well studied in this context (e.g [7, 10]), much less is known about how semiaquatic mammals respond to habitat fragmentation, applying also to our study species, the Iberian desman [8].

The Iberian desman, *Galemys pyrenaicus*, is the only species of its genus and is sister to another semiaquatic species, the Russian desman, *Desmana moschata*. Together these two species form the tribe Desmanini within the family Talpidae (the moles and related taxa; [11, 12]). The Iberian desman is endemic to southwestern Europe, where it is restricted to the Pyrenees, northern and central Spain, and northern Portugal, and is listed as Endangered by the IUCN [11]. Over recent decades, the Iberian desman has experienced a severe decline in its range, retreating to cooler, mountainous headwater streams [8, 13]. The species is considered one of the rarest and least-studied European mammals [14]. The limited amount of research on such a rare species undergoing a steep decline over all its range raises concerns about undetected population isolation and inbreeding, potentially putting populations at risk.

In relation to the genetic studies that have been carried out on the Iberian desman, the first detailed phylogeographic analysis on the species detected low levels of genetic diversity and identified four distinct mitochondrial lineages [15]. Similar geographic structuring was found with SNP data from double digest Restriction-site Associated DNA sequencing (ddRAD), but instead describing five distinct phylogeographic units (Occidental, Central System, Iberian Range, Pyrenees, Cantabria) [16]. These units show limited admixture between them and are largely coincident with the main mountain ranges where the Iberian desman occurs, with the exception of the Occidental phylogeographic unit (with a large range in northern Portugal and Galicia). While these broader patterns reflect major phylogeographic entities separated by low connectivity, local demography and dispersal patterns need to be defined within phylogeographic units to further understand population structure in the species. Recent studies suggest that the decline in Iberian desman populations may lead to high inbreeding levels and local extinctions in the Pyrenees [17] and in the Iberian Range [18]. ddRAD and relatedness estimates based on tissue samples of individuals from nearby rivers of the Iberian Range have also shown that there is lower dispersal between rivers compared to within rivers [18]. Clearly the degree of dispersal between nearby rivers is important for connectivity within geographic regions [19].

So far, genomic studies on the Iberian desman have provided limited information on the Occidental and Central System phylogeographic units. It is known that populations from the Occidental phylogeographic unit exhibit the highest levels of heterozygosity [16, 20] and nucleotide diversity [15], whereas those from the Pyrenees and Central System show the lowest levels [15–17, 20]. This suggests that the Occidental phylogeographic unit may have served as a major glacial refugium for desman populations during the Last Glacial Maximum [15], being a key reservoir of genetic diversity for this species, underscoring its conservation value. This is particularly important considering that the Iberian desman is among the most inbred and genetically least variable mammals assessed to date [17, 20]. Maintaining the genetic variation within the Occidental phylogeographic unit may enhance the species’ adaptability to changing environments, providing resilience to various threats. As such, the Occidental phylogeographic unit is not only important for conservation in its own right, but also a potential source for genetic rescue for other phylogeographic units [21].

Previous research, using the cyt*b* mitochondrial marker, provided some insights into the genetic structure and dispersal patterns of the Iberian desman in the Occidental phylogeographic unit, especially across the Douro and Minho river systems [19]. These existing analyses suggested that, overall, overland movements played an important role in postglacial colonization, while within a smaller basin (the Minho) both river and overland dispersal have contributed to gene flow. However, these conclusions were based mainly on large-scale isolation-by-distance patterns within the phylogeographic unit. Building on this foundation, the research that we report here addresses instead the microgeographic genomic variation in the Occidental phylogeographic unit of the Iberian desman, focussing on populations from two adjacent watersheds in Portugal (part of the Douro river system), where they occur in the upper reaches. These watersheds allowed us to test two key aspects of desman dispersal and connectivity at a fine scale: (1) Linear/river connectivity within watersheds – evaluating movement and gene flow along the river, and (2) Dispersal across headwaters – assessing movement and gene flow between adjacent watersheds.

To address these questions, we applied ddRAD sequencing to non-destructive tissue samples from two adjacent watersheds within the Occidental phylogeographic unit. This high-resolution genomic approach provides new data from this genetically diverse phylogeographic unit, enabling us to assess fine-scale dispersal and connectivity patterns that are critical for conserving the Iberian desman in fragmented riverine systems and for maintaining its adaptive potential under future environmental change.

## Methods

### Sample collection

Non-destructive Iberian desman tissue samples were collected between 2015 and 2016 in Northern Portugal in the scope of a wider ecological study on the species by the LTER Network [8], in the Douro river system, at the Baixo Sabor Long Term Ecological Research Site (LTER Network – http://lternet.edu) and nearby areas (see Quaglietta et al. [8] for more details about sample collection). The watersheds involved were the Sabor and Tua (N 41°09′–42°06′, W 7°37′–6°16′). Additionally, two tissue samples from the Minho river system (2012) and two from the Ulla River system (2013), both from Galicia, Spain, were collected and provided by ARCEA Xestión de Recursos Naturais S.L. For the collection of tissues, sterilized tweezers were used, and samples were kept in 96% ethanol until DNA extraction. Individual desmans providing samples were georeferenced in the field using a GPS receiver. A total of 15 tissue samples from Portugal and Spain were used to extract DNA (Additional File 1 - Table S1).

Tissue availability was limited, as we relied on previously collected samples obtained for other purposes. Given the endangered status of the species, collecting new material is extremely difficult, which constrains the number of samples that can be included in genomic analyses.

### ddRAD protocol including DNA extraction, PCR amplification and sequencing

ddRAD sequencing was applied to the non-destructive tissue samples. As a genomic methodology, ddRAD produces a substantial number of SNP markers, permitting detailed analysis of genetic diversity and population structure, as demonstrated in previous conservation genetic studies (e.g [22]). This genomic approach uses two restriction enzymes enabling paired-end sequencing at specific loci across many samples, offering greater precision in read mapping compared to genotyping by sequencing (GBS) or single-enzyme RAD sequencing (RADseq) [23, 24].

For each individual, DNA was extracted using the Qiagen DNeasy Blood & Tissue extraction kit, following the manufacturer’s instructions. DNA quantification was assessed using a Qubit 2.0 Fluorometer. DNA libraries were constructed following the protocol of Peterson et al. [24] with modifications. Genomic DNA (~ 150 ng) was digested with three units of SbfI-HF and six units of MspI, while simultaneously ligating P1/SbfI (sample barcode) and P2/MspI adapters with 120 cohesive end units of T4 DNA ligase. Reaction volumes were 30 µl in 1× CutSmart buffer and 1 mM ATP. All primers were from Integrated DNA Technologies; all other reagents were from New England Biolabs. Following digestion/ligation, 1.5 µl of each sample was amplified in a 10 µl volume with 2.5 pmol Illumina Truseq P1 (AATGATACGGCGACCACCGAGATCTACACTCTTTCCCTACACGACGCTCTTCCGAT) and index (CAAGCAGAAGACGGCATACGAGATxxxxxxGTGACTGGAGTTCAGACGTGTGC, where xxxxxx is the index group sequence) primers, 0.2 mM dNTPs, and 1.25 units One Taq DNA polymerase. PCR reactions were cycled at 95 °C for 40 s, 60 °C for 45 s, and 68 °C for 30 s, for 27 cycles. Sample aliquots were pooled and small fragments were removed with Ampure XP (0.7×). The library was diluted to 2nM and sequenced in a single lane on an Illumina HiSeq 2500 platform (paired-end 155 bp reads), producing an average of 2,322,237 reads per sample.

### Data analysis

The repetitive regions in the reference genome available in NCBI (Gpyr_1.0 - GCA_019455555.1 [20]) were predicted and masked by RepeatMasker v4.1.4 (http://www.repeatmasker.org) using the Dfam Consensus release 20,220,412 database [25].

#### Species-unit assignment

We first verified whether the 15 Iberian desmans that we sequenced from the western part of the Iberian Peninsula clustered as expected within the previously-identified Occidental phylogeographic unit [15, 16, 19, 20]. For that, we combined our newly generated data with publicly available datasets, which include 94 individuals sequenced using ddRAD and 6 individuals with whole-genome sequences, distributed over the majority of the species’ range (Additional File 1 - Table S1). Our strategies to analyse the combined dataset reflect our use of different restriction enzymes to generate the ddRAD data than those used by the previous researchers. A quality distribution plot of the read data for the 115 samples was generated using FASTQC [26]. The combined dataset was trimmed and adapters removed with AdapterRemoval v2.3.2 [27] and then aligned to the reference genome (GCA_019455555.1 - [20]) using BWA-MEM with default parameters [28]. For the whole-genome data, Picard v3.3.0 with the *MarkDuplicates* command (https://github.com/broadinstitute/picard) was used to identify and remove duplicate reads (Additional File 1 – Table S2). To build and genotype the paired-end data and call SNPs, *bcftools mpileup* (with the --*min-MQ 30* and --*min-BQ 30* commands, to consider only reads and bases with quality above 30) and *bcftools call* (with the -*-mv* command) was used. After obtaining the vcf file, *bcftools* was used to filter variants, keeping sites with either a quality score above 30 or a read depth greater than 500 in at least one sample (with the *filter -i ‘QUAL >30 || FORMAT/DP >500’* command). Then we kept the SNPs that were present in our samples to minimize missing data using the --*positions* command from *vcftools*, since we wanted a compatible subset of SNPs. After that, *vcftools* was again used to remove variants with more than 80% of missing data (*--max-missing 0.8* command), remove individuals with more than 40% of missing data (--*missing-indv* command to check missingness per individual, followed by --*remove*), keep only biallelic SNPs (*--max-alleles 2* command), and retain only SNPs where the minor allele was present at a frequency of at least 5% (*--maf 0.05* command) (Additional File 1 – Table S2). Finally, a Principal Component Analysis (PCA) was made using the *--pca* command from PLINK v1.9 [29]. To check phylogenetic relationships between phylogeographic units, the dataset was bootstrapped 100 times to ensure statistical power using a custom script (https://github.com/pdroslva84/Plink_IBS_bootstraps) and a neighbour-joining tree was constructed using PHYLIP v3.6 [30] with 100 bootstraps, using the Russian desman (*Desmana moschata*) as outgroup (GCA_965120235 - [31]). The raw sequencing reads from *D. moschata* (ERR13337211) were downloaded and processed identically to the Iberian desman samples, and genotypes were then filtered according to the same criteria as for all other samples (above).

#### Microgeographic genomic analysis in the Occidental phylogeographic unit

After confirming that the 15 samples clustered within the Occidental phylogeographic unit and having supported the existence of five main units in the Iberian Peninsula, we conducted a detailed analysis of our new samples. A quality distribution plot of the read data of the 15 samples was generated using FASTQC [26]. Demultiplexing by barcode was carried out using the *process_radtags* utility included in Stacks 2.62 package [32] for our 15 newly generated samples, because they were multiplexed in the same sequencing lane (the publicly available dataset in the previous section was already available as individual FASTQ files per sample, so no demultiplexing was needed for those). After demultiplexing, adapter removal and quality filtering were applied uniformly to all samples except this time *bcftools* was used to filter variants, keeping sites with either a quality score above 30 or a read depth greater than 70 in at least one sample. Also *vcftools* was used to remove variants with more than 90% missing data (*--max-missing 0.9* command) and remove individuals with more than 20% of missing data (--*missing-indv* command to check missingness per individual, followed by --*remove*), retaining only biallelic SNPs (*--max-alleles 2* command) with a minor allele frequency (MAF) of at least 5% (*--maf 0.05* command). While a 5% MAF filter may seem high for 15 individuals, this threshold ensures that variants are present in at least two heterozygous individuals (or one homozygote), avoiding single-allele occurrences and reducing noise from rare or spurious variants. Before proceeding with the analysis, we first checked if there were any related samples using the *--relatedness2* command from *vcftools*, using a threshold above 0.5. The details of SNP and sample retention after the filtering steps are given in Additional File 1 – Table S3. A Principal Component Analysis (PCA) was performed using the *--pca* command from PLINK v1.9 [29] and a Discriminant Analysis of Principal Components (DAPC) was made using the *adegenet* package from R [33]. Population structure was estimated with the program STRUCTURE 2.3.4, which implements a Bayesian model-based clustering method [34], with no prior information on population origin. A total of 100,000 generations were run after a burn-in of 10,000 generations with a number of clusters (K) ranging from 1 to 8. We implemented 3 different runs for each K value in order to evaluate the variance of the estimated posterior probability of the data, Ln P(D) [34]. In addition, the optimal K value was estimated using the ΔK method [35] using CLUMPAK [36]. Genetic distances between individuals were calculated using the *--distance* function of PLINK v1.9 and the *--1-ibs* and *--square* modifiers. A PERMANOVA (Permutational Multivariate Analysis of Variance; [37]) using the *adonis2* from the *vegan* package from R [38] was used to test for significant variation in genetic distance comparisons between and within river systems. To test for isolation-by-distance (IBD), the correlation of genetic distances and Euclidean (overland) distances, Mantel tests [39] were performed in R (using the *vegan* package with the *mantel* command, applying the “spearman” method and 9999 permutations). For the Douro dataset we also conducted Mantel tests using river distances rather than overland distances, making use of a river network geodatabase for Europe [40] and the *sf* [41] and *sfnetwork* [42] R packages. To account for movement between watersheds on this same dataset, we also carried out partial Mantel tests (*vegan* package, *mantel.partial* command, “pearson” method and 999 permutations), to check the effect of overland distance on genetic distance while controlling for river distance and vice versa. To visualize these relationships, we produced scatterplots in R with the *ggplot2* package [43] of pairwise genetic distances against both overland and river distances. Pairs of individuals within the Sabor watershed, within the Tua watershed, and across the two watersheds were shown in different colours, and linear regression lines were added for each group. These plots allow direct comparison of how river and overland distances relate to genetic differentiation within and across watersheds.

An overview of the workflow used in this study is illustrated in Additional File 2 - Fig. S1. The diagram depicts the sequence of descriptive, statistical and computational tests conducted to examine population structure and connectivity in the Iberian desman.

## Results

### Phylogeographic subdivision in the Iberian desman

**Fig. 1:**
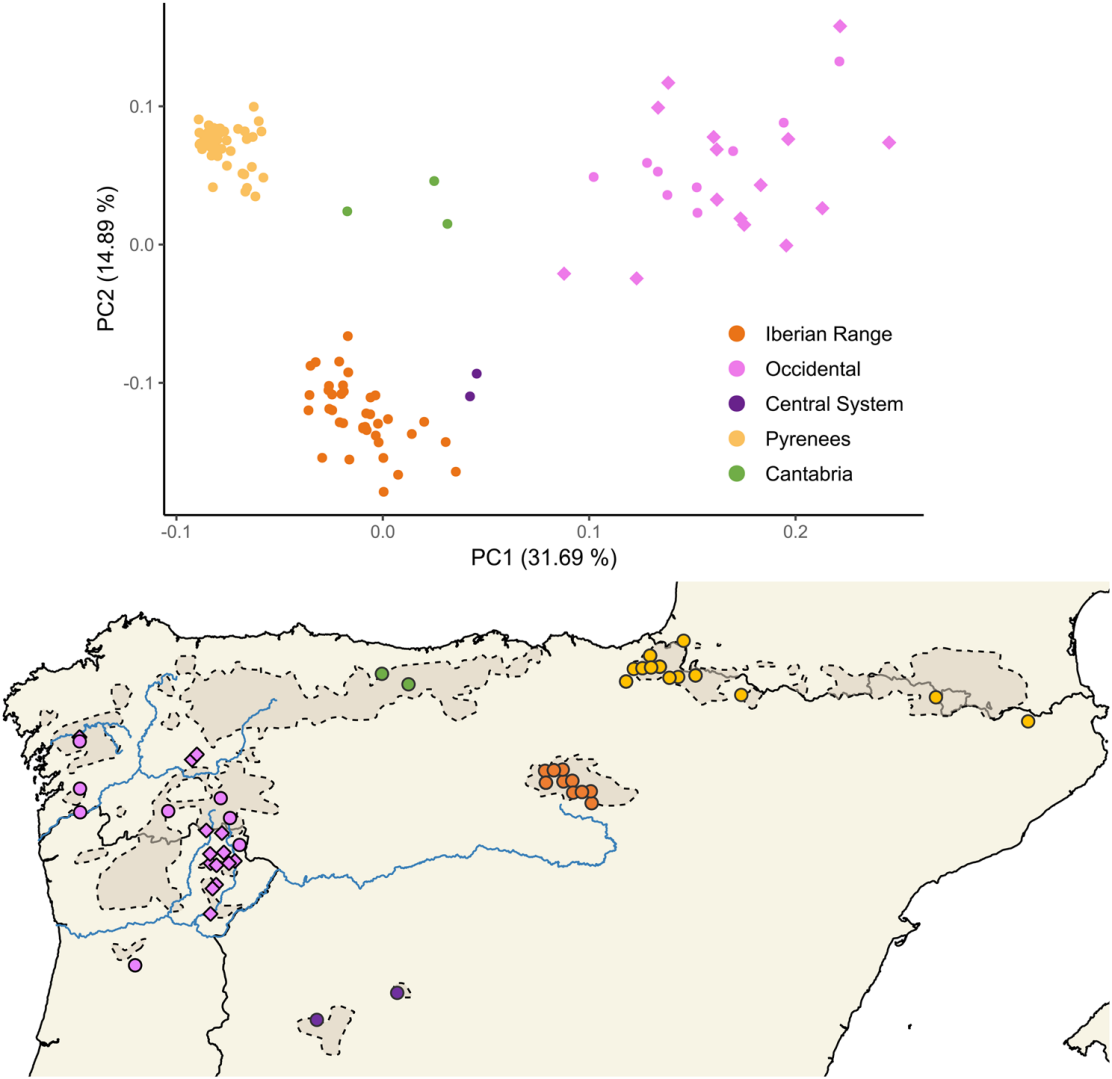
Principal component analysis and map containing data from 115 samples of the Iberian desman, *Galemys pyrenaicus*, showing the five main phylogeographic units from the Iberia Peninsula (Occidental, Central System, Cantabria, Iberian Range and Pyrenees - [16]) based on 110 SNPs. Diamonds represent our new samples. Dashed lines with darker shading delimit the current species’ distribution according to Quaglietta et al. [11]. From north to south, the Ulla, Minho and Douro river systems are also shown, with the Tua and Sabor rivers as tributaries of the Douro

For the complete dataset of 115 Iberian desmans for which genomic data were available, a total of 110 SNPs were obtained. This reduced number of SNPs reflected the use of different restriction enzymes across the combined ddRAD datasets. The PCA based on these SNPs shows five major phylogeographic units (Occidental, Central System, Cantabria, Iberian Range, Pyrenees) as described in Querejeta et al. [16] (Fig. 1). Our new samples clustered within the Occidental phylogeographic unit.

The neighbour-joining tree (Additional File 2 - Fig. S2) further supports these five phylogeographic units, emphasizing divergence of the Occidental phylogeographic unit from the others (although with low bootstrap support: 58). The Pyrenees phylogeographic unit also forms a relatively distinct cluster (bootstrap support: 78).

While the reduced dataset cannot resolve fine-scale structure, it was used as a reference to place our new samples within their broad geographic context, confirming their assignment to the Occidental phylogeographic unit.

### Microgeographic genomic analysis in the Occidental phylogeographic unit

After confirming that our newly generated dataset belongs to the Occidental phylogeographic unit, we conducted a microgeographic analysis using a larger SNP panel to investigate patterns of genetic variation within this unit.

**Fig. 2:**
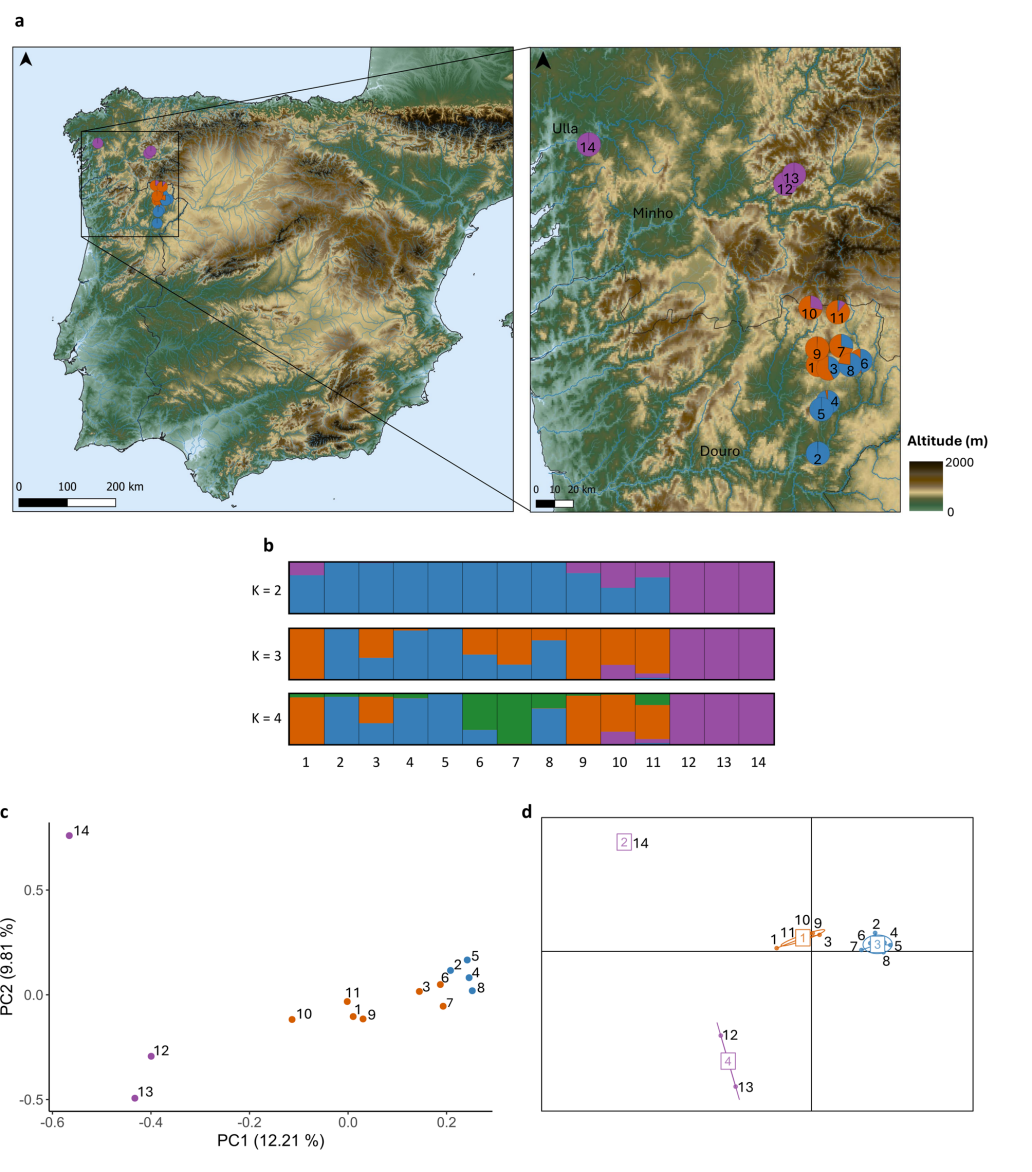
Genetic and geographic analysis of our ddRAD data (*N* = 14, 7,604 SNPs) with colouring of sample points to emphasise similarities and differences: **(a)** sample locations on a topographical background, with pies indicating the best-supported STRUCTURE results for each individual (see Fig. 3 for a higher resolution depiction of samples collected along the Tua and Sabor rivers); **(b)** STRUCTURE plot showing clusters for K = 2, K = 3 and K = 4, where the best-supported K is 3 (Additional File 2 - Fig. S5); **(c)** Principal Component Analysis results with colours reflecting the STRUCTURE results; **(d)** Discriminant Analysis of Principal Components results using the best-supported value of 4 clusters (Additional File 2 - Figs. S3 and S4 and Additional File 1 - Table S6) and with colours reflecting the STRUCTURE results

One of our samples (from Riobó) was removed (sample 15), because of its close relatedness (*r* = 0.58) to another. For the 14 remaining samples, we obtained 7,604 SNPs. The genetic distances between individuals ranges from 0.2247 to 0.3196 (Additional File 1 - Table S4a) and the overland distances between them ranges from 5 to 207 km (Additional File 1 - Table S5a). PCA, DAPC and STRUCTURE analyses showed clear geographic clustering of the samples (Fig. 2). PCA and DAPC show similar results, with DAPC showing the best K as 4 (Additional File 1 - Table S6; Additional File 2 - Figs. S3 and S4), but also separating the northernmost samples (12, 13 and 14) along the first axis of variation (Fig. 2a and b). The same pattern was observed in STRUCTURE, despite the best K being 3 using the ΔK method (Evanno et al. 2005) (Additional File 2 - Fig. S5). PERMONOVA demonstrated that the genetic differences between the northernmost samples and the remaining ones were significant (Additional File 1 - Table S7a). The Mantel test applied to all 14 samples showed significant isolation-by-distance (IBD) with a strong correlation between genetic and overland distances (*r* = 0.79, *p* = 0.0001 - Additional File 1 - Table S8).

**Fig. 3:**
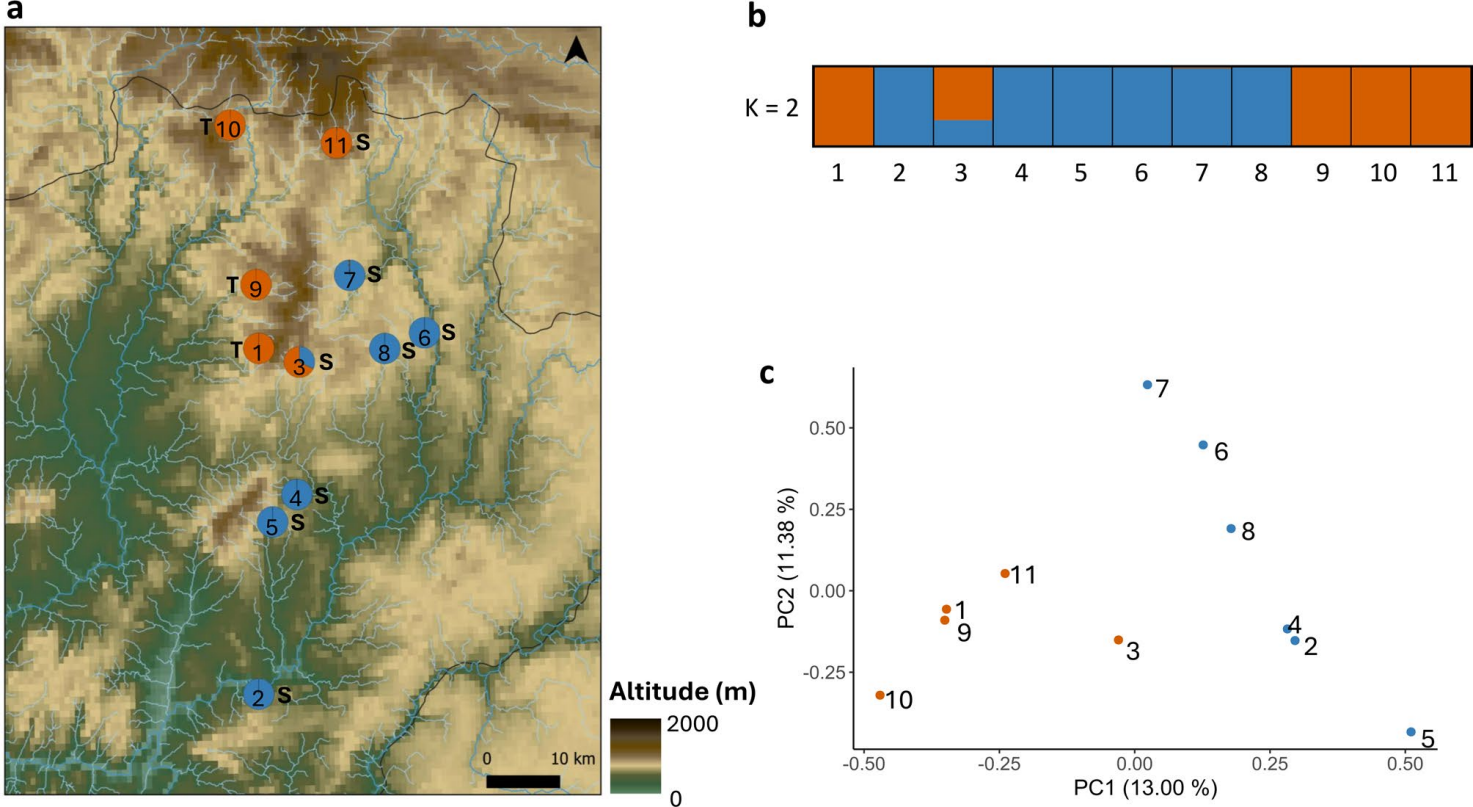
Genetic and geographic analysis of ddRAD data (*N* = 11, 8,771 SNPs) with colouring of sample points to emphasise similarities and differences: (**a**) sample locations on a topographical background, with pies indicating the best-supported STRUCTURE results for each individual, while depicting Sabor (S) and Tua (T) watersheds; (**b**) STRUCTURE plot showing clusters for K = 2, where the best-supported K is 2 (Additional File 2 - Fig. S6); (**c**) Principal Component Analysis results from the two subpopulations from the Douro river system, and colours reflecting the STRUCTURE results

A hierarchical approach was used to examine genetic structure in the Douro river system, considering 11 samples from two watersheds (Tua and Sabor) and excluding the 3 Galician samples (12, 13 and 14) which are from different river systems (Minho and Ulla; Fig. 2a). A total of 8,771 SNPs were available. The genetic distances between individuals ranges from 0.2122 to 0.2744 (Additional File 1 - Table S4b) and the overland and river distances between them ranges from 5 to 79 km and from 21 to 309 km, respectively (Additional File 1 - Table S5b). Both STRUCTURE (Fig. 3a and b) and PCA (Fig. 3c) analyses showed geographic clustering of individuals, with STRUCTURE showing the best K as 2 using the ΔK method (Evanno et al. 2005) (Additional File 2 - Fig. S6). Moreover, there is genetic differentiation by watershed, as shown in the PERMANOVA analysis, which demonstrated significant differences between the samples from each watershed (Additional File 1 - Table S7b). Nevertheless, there are also indications of genetic exchange between the headwaters, since individuals 3 and 11 cluster with Tua samples despite being from the Sabor watershed (Fig. 3a). The Mantel test for the 11 samples did not reveal IBD (*p*-value > 0.01) when using overland distances (*r* = 0.34, *p* = 0.02 – see Additional File 1 - Table S8), though it revealed IBD when using river distances (*r* = 0.42, *p* = 0.003). Considering the possibility of terrestrial dispersal between headwaters, partial Mantel tests were also made to compare the effects of overland and river distances on genetic differentiation (Additional File 1 - Table S8). These tests yielded very similar results comparing to the previous Mantel tests (overland: *r* = 0.34, *p* = 0.018 controlling for river; river: *r* = 0.42, *p* = 0.002 controlling for overland), indicating that controlling for one distance type hardly reduced the correlation with the other. This suggests that river and overland distances explain largely independent components of genetic differentiation in this two-watershed system, which is consistent with gene flow occurring both along rivers and via short overland dispersal between adjacent headwaters. The same pattern is also illustrated in the scatterplots comparing genetic with geographic distances. Genetic differentiation increases with both overland and river distance in both the Tua and Sabor watersheds (Additional File 2 - Fig. S7a and b). Across watersheds, genetic distances were generally higher than within basins but overlapped substantially, suggesting some connectivity. The regression of genetic distance on overland distance across watersheds is positive while the regression of genetic distance on river distance is negative, indicating that genetic similarity is not constrained by the long riverine paths separating watersheds and is instead likely mediated by short overland routes between the headwaters (see also Additional File 2 - Fig. S7c). Thus, these results are consistent with both river-mediated and short-distance overland dispersal.

## Discussion

### Phylogeographic subdivision in the Iberian desman

The newly generated SNP data, combined with the compatible subset of SNPs from published data, distinguishes the same five major phylogeographic units of the Iberian desman as described in Querejeta et al. [16] (Fig. 1). This broad phylogeographic pattern was detected using 110 SNPs. Although there were a low number of SNPs, due to the different restriction enzymes used between studies, the dataset is still informative for broader phylogeographic comparisons and demonstrates the utility of datasets with small numbers of genetic markers, a limitation that may often be present in low budget conservation genomic studies and when there is low genetic diversity in rare and limited range species. Reduced SNP datasets have been successfully utilised in other population studies on various taxa (e.g [22, 44–46]).

The small SNP dataset also generated interesting phylogenetic results, although with relatively low bootstrap support (Additional File 2 - Fig. S2). Unlike previous phylogenetic analyses of the Iberian desman using mitochondrial data [15, 20], which cluster the Pyrenees, Cantabrian, and northwest Iberian Range phylogeographic units into one group and the southeast Iberian Range, Central System, and Occidental phylogeographic units into another, our SNP-based analysis does not show the Iberian Range phylogeographic unit split into two clades. The neighbour-joining tree separates the Occidental phylogeographic unit from the remaining phylogeographic units, although with bootstrap support of only 58. This separation may reflect historical isolation of the Occidental phylogeographic unit, perhaps due to its location in a glacial refugium during the Pleistocene. The Pyrenees phylogeographic unit forms a distinct cluster with stronger bootstrap support (78). Igea et al. [15] posited that this population likely originated from a distant glacial refugium in the Basque Mountains. They suggested that the population in the Pyrenees colonized this region relatively recently, following a severe bottleneck. Escoda and Castresana [20] further emphasized extremely low genomic diversity and high levels of inbreeding in Pyrenean populations, likely due to repeated bottlenecks during postglacial recolonization. Taken together, these contrasting phylogenetic signals underline the need for further analysis to determine the basis of the differences between the findings with mitochondrial and nuclear data. While low bootstrap values limit definitive conclusions regarding finer-scale relationships, our neighbour-joining tree supports the overall phylogeographic units established by Querejeta et al. [16]. Nevertheless, the limited number of SNPs indicates that these results should be interpreted with caution. They are robust in confirming the main phylogeographic divisions and validating the placement of our newly generated dataset within the Occidental phylogeographic unit, but they cannot resolve more detailed or recent evolutionary relationships.

### Microgeographic genomic analysis in the Occidental phylogeographic unit

Unlike the reduced number of SNPs available in the combined dataset due to the use of different restriction enzymes across studies, the Occidental dataset alone yielded more than 7,000 SNPs, providing a robust basis for fine-scale population analyses.

The 14 desman samples analysed from the Occidental phylogeographic unit (using 7,604 SNPs) showed genetic separation of the three from northernmost Galicia (12, 13 and 14) (Fig. 2; Additional File 1 - Table S7a). This likely indicates their origin from different river systems than the Douro, and therefore reflects differences in their recent evolutionary history.

The Mantel test for the 14 samples that we analysed showed a significant positive correlation between genetic and overland distance, thus suggesting IBD (Additional File 1 - Table S8). This indicates that gene flow decreases with increasing geographic distance and that dispersal occurs primarily over short distances, a pattern typical of species with constrained movement capabilities (as would be expected for a small mammal). These findings at a larger scale align with earlier studies [19, 47–49], which revealed limited dispersal in the Iberian desman. Similar IBD patterns have been observed in studies of other taxa [50, 51], including other semiaquatic mammals such as otters [52], where restricted dispersal and habitat specialization contribute to localized genetic differentiation. These findings highlight the importance of local connectivity for maintaining genetic exchange within fragmented habitats.

The IBD observed for the 14 samples may substantially be the consequence of the distinctiveness of the three Galician samples, distantly located from other samples. The degree of genetic structuring within the Douro river system of the 11 samples from the Tua and Sabor watersheds (8,771 SNPs) was therefore of particular interest. The desmans from the Tua and Sabor watersheds are genetically distinct from each other (Additional File 1 - Table S7b) but our data also indicate dispersal between the watersheds across adjacent headwaters, as exemplified by the individuals 3 and 11, which cluster with Tua samples despite being from the Sabor river, and also by the results from the partial Mantel tests (Additional File 1 - Table S8) and the scatterplots (Additional File 2 – Fig. S7). Together, our findings suggest that dispersal within basins is largely shaped by riverine distance, while across watersheds it is facilitated by short overland routes at the headwaters. Similar reliance on terrestrial pathways to cross between fragmented aquatic habitats has been observed in other organisms [9]. These findings partially align and contrast with previous mitochondrial data from Querejeta et al. [19]. They analysed 157 samples (44 from Minho, 55 from Douro and 58 from other river systems) using cyt*b* and D-loop genes (a total of 1,066 bp) and found a strong IBD signal at the regional scale (i.e., their whole study area). Similarly, we also observed strong IBD between genetic and overland distances in our analysis of 14 samples (*r* = 0.79, *p* = 0.0001). For the Douro river system, the same authors reported a weak IBD. Our findings showed a significant and strong correlation between genetic and river distances in both Mantel and partial Mantel tests (*r* = 0.42, *p* = 0.003; *r* = 0.42, *p* = 0.002). Moreover, scatterplots illustrate that river distances align more closely with genetic differentiation within each watershed, whereas overland distances provide a better fit for the across watersheds comparison, consistent with both river-mediated and short-distance overland dispersal. This and the partial Mantel tests suggest that even though rivers are important for movement and gene flow, especially for semiaquatic species like the Iberian desman, both overland and riverine routes might play important roles in shaping its genetic connectivity. The non-significant IBD for the Douro river system reported by Querejeta et al. [19] could be explained by the fact that they did not capture the effects of microgeographic dispersal dynamics and instead their results reflected large-scale differentiation within this river system (i.e., without accounting for closely located headwaters).

While limited inter-river dispersal has been previously observed [18], our study provides evidence of such dispersal between the upper reaches of closely situated rivers, and is the first report of this kind within the Occidental phylogeographic unit. Our findings highlight the importance of headwaters in maintaining genetic connectivity, counteracting the isolation observed downstream where larger river systems often show suboptimal conditions for dispersal (as already mentioned by Escoda et al. [18]). These headwater habitats not only maintain genetic exchange between watersheds but also provide critical refuges that buffer against environmental change, in particular in current climate change scenarios [53]. Other semiaquatic vertebrates show similar results, with the upper reaches of rivers acting as genetic reservoirs supporting metapopulations across fragmented landscapes [7].

Our microgeographic genomic study not only enhances our general understanding of habitat fragmentation and dispersal in the Iberian desman, it also provides new genetic insights into the Occidental phylogeographic unit, highlighting the role of dispersal between watersheds in maintaining genetic diversity in this system. In particular, given its high genetic diversity compared to the other phylogeographic units [15, 16, 20], the Occidental phylogeographic unit could serve as an important source population for genetic rescue efforts aimed at mitigating the risks of inbreeding and genetic drift in vulnerable populations in other parts of the distribution of the Iberian desman. Examples in other species where relatively genetically diverse populations have acted as a source in genetic rescue of threatened populations include the mountain pygmy possum [54], the Allegheny woodrat [55] and the brook trout [56].

## Conclusion

Our study highlights the genetic subdivision of the Iberian desman, confirming the existence of five major phylogeographic units, supporting the hypothesis of post-glacial isolation and then expansion although with limited recent connectivity among phylogeographic units. Additionally, the use of a small SNP dataset to uncover broader genetic structure demonstrates a cost-effective approach for studying endangered species in resource-limited settings. However, the restricted number of SNPs also constrains the resolution of the analyses, and the results should therefore be interpreted with caution.

With a higher-resolution SNP dataset, our genomic analysis revealed isolation-by-distance in the Occidental phylogenetic unit, as would be expected for a small mammal with limited dispersal. Furthermore, our spatial microgeographic analysis in the Douro river system showed riverine connectivity but also overland dispersal between adjacent headwaters, especially across short terrestrial routes. This latter finding corroborates that headwater connectivity, both aquatic and terrestrial, plays a critical role in facilitating gene flow within fragmented riverine systems.

From a conservation perspective, these findings reinforce the importance of preserving both riparian and terrestrial corridors between adjacent headwaters. In particular, for the Iberian desman Occidental phylogenetic unit headwaters serve as critical pathways for gene flow, mitigating the isolation imposed by downstream habitat fragmentation. Protecting these upland areas is essential for preserving the genetic diversity of the species, which provides the basis for its adaptability and long-term survival in a rapidly changing environment. High genetic diversity may be important not only to conserve the Occidental phylogenetic unit itself, but to provide opportunities for genetic rescue of vulnerable populations in other phylogenetic units of the desman.

Future research should expand on our findings by including larger sample sizes, additional watersheds, and higher-resolution genomic datasets to better document the genetic diversity of the Iberian desman over the whole Occidental phylogenetic unit and, in particular, to understand the interplay between aquatic and terrestrial connectivity within this system. Likewise, telemetry studies would provide further complementary knowledge on fine-scale dispersal routes and behaviour within and between rivers. Such efforts will be crucial for developing targeted conservation strategies that enhance connectivity across fragmented landscapes, ensuring the Iberian desman’s persistence and the maintenance of its genetic health.

## Supplementary Information

Below is the link to the electronic supplementary material.

Supplementary Material 1: Table S1: Details of the 115 samples used in this study, including the accession number from INSDC (when available – the others were retrieved from DRYAD), locality, phylogeographic unit, latitude, longitude, sex, and year of collection. For the Occidental phylogeographic unit samples collected in this study, additional information is provided on watershed of origin. Table S2: Details of the 115 samples used in this study, including the total number of raw reads, mapping rate (as a percentage), duplication rate (as a percentage) and mean coverage for each sample before filtering, and number of SNPs, mean depth and the proportion of sites that are missing in a sample after *bcftools* (BT) and *vcftools* (VT) filtering steps. Table S3: Details of the samples used in this study from the Occidental phylogeographic unit, including the number of SNPs, mean depth and the proportion of sites that are missing in a sample after *bcftools* (BT) and *vcftools* (VT) filtering steps, for all the 15, 14 (Occidental), 11 (Douro) and 8 (Sabor) samples. Table S4: Genetic distances between individuals (see text for further details): (a) for the Occidental phylogeographic unit; (b) for the Douro river system; (c) for the Sabor watershed. Table S5: Matrix of overland (dark green) and/or river (dark blue) distances between samples, in meters: (a) for the Occidental phylogeographic unit; (b) for the Douro river system; (c) for the Sabor watershed. Table S6: Discriminant Analysis of Principal Components posterior probabilities for the Occidental phylogeographic unit, taking 6 principal components into account: (a) for 2 groups; (b) for 3 groups; (c) for 4 groups. Highlighted cells indicate the samples assigned to each cluster (K) based on their highest posterior probability. Table S7: Permutational Multivariate Analysis of Variance results testing genetic differences between samples on a genetic distance matrix: (a) for the Occidental phylogeographic unit (Douro vs. other river systems); (b) for the Douro river system (Tua vs. Sabor watersheds). Df stands for degrees of freedom, SS for sum of squares, R2 for proportion of variation explained and F for F-statistic. Values with *p* < 0.01 are highlighted in bold. Table S8: Mantel test using genetic distances and overland and river distances for the Occidental phylogeographic unit, Douro river system and the Sabor watershed showing the correlation coefficient (r) and *p*-value for each test. Values with *p* < 0.01 are highlighted in bold. Missing values (-) are due to differences in the spatial context of the analyses. For the Occidental phylogeographic unit, river distances were not calculated because the samples are distributed across different river systems, making direct river-based distances biologically meaningless. To disentangle the relative contributions of overland and river distances for the Douro river system, partial Mantel tests were performed: (i) testing the effect of overland distance on genetic distance while controlling for river distance (Overland distances), and (ii) testing the effect of river distance on genetic distance while controlling for overland distance (River distances).

Supplementary Material 2: Fig. S1: Diagram showing the flow of analyses for this manuscript. Fig. S2: Neighbour-joining tree based on the 115 samples and 110 SNPs using PHYLIP v3.6 with 100 bootstraps and the Russian desman (*Desmana moschata*) as outgroup. Bootstrap values from 100 replicates are displayed for nodes with support ≥ 50%. The five phylogeographic units are highlighted. Fig. S3: Bayesian Information Criterion (BIC) values for each number of clusters for the Discriminant Analysis of Principal Components analysis taking 6 principal components into account for the samples from the Occidental population, based on 7,604 SNPs. Fig. S4: Discriminant Analysis of Principal Components for the Occidental phylogeographic unit, with 6 principal components and based on 7,604 SNPs, showing 3 clusters. Fig. S5: STRUCTURE v2.3.4 analyses for the Occidental phylogeographic unit, based on 7,604 SNPs: (a) the rate of change in the likelihood of the data as K increases (Delta K) by Evanno et al. [1]; (b) the probability or likelihood of the data for each K (Prob(K)) based on the Bayesian clustering algorithm by Pritchard et al. [2]. Fig. S6: STRUCTURE v2.3.4 analyses for the Douro river system, based on 8,771 SNPs: (a) the rate of change in the likelihood of the data as K increases (Delta K) by Evanno et al. [1]; (b) the probability or likelihood of the data for each K (Prob(K)) based on the Bayesian clustering algorithm by Pritchard et al. [2]. Fig. S7: Scatterplots of genetic distance against geographic distances in the Douro river system. (a) Genetic distance versus overland distance, (b) genetic distance versus river distance, and (c) overland versus river distance. Each point represents a pair of individuals, coloured by watershed: Sabor (orange), Tua (blue), and across watersheds (green). Linear regression lines are shown for each category. These plots illustrate that river distances align more closely with genetic differentiation within watersheds, whereas overland distances provide a better fit for between-watershed comparisons, consistent with both river-mediated and short-distance overland dispersal.

## Data Availability

The ddRAD data generated in the present study has been deposited in the European Nucleotide Archive (https://www.ebi.ac.uk/ena/browser/view/PRJEB88519). Details of all data generated or analysed during this study are included in this published article and its supplementary information files.
