# Supplementary material for: Microgeographic genomic variation and connectivity in an endangered semiaquatic mammal
Source: BMC Ecol Evol. 2025 Oct 23;25:114. doi: 10.1186/s12862-025-02460-1 (PMC12548277; doi:10.1186/s12862-025-02460-1)
Supplement: Supplementary file 1 — Supplementary Material 1: Table S1: Details of the 115 samples used in this study, including the accession number from INSDC (when available – the others were retrieved from DRYAD), locality, phylogeographic unit, latitude, longitude, sex, and year of collection. For the Occidental phylogeographic unit samples collected in this study, additional information is provided on watershed of origin. Table S2: Details of the 115 samples used in this study, including the total number of raw reads, mapping rate (as a percentage), duplication rate (as a percentage) and mean coverage for each sample before filtering, and number of SNPs, mean depth and the proportion of sites that are missing in a sample after bcftools (BT) and vcftools (VT) filtering steps. Table S3: Details of the samples used in this study from the Occidental phylogeographic unit, including the number of SNPs, mean depth and the proportion of sites that are missing in a sample after bcftools (BT) and vcftools (VT) filtering steps, for all the 15, 14 (Occidental), 11 (Douro) and 8 (Sabor) samples. Table S4: Genetic distances between individuals (see text for further details): (a) for the Occidental phylogeographic unit; (b) for the Douro river system; (c) for the Sabor watershed. Table S5: Matrix of overland (dark green) and/or river (dark blue) distances between samples, in meters: (a) for the Occidental phylogeographic unit; (b) for the Douro river system; (c) for the Sabor watershed. Table S6: Discriminant Analysis of Principal Components posterior probabilities for the Occidental phylogeographic unit, taking 6 principal components into account: (a) for 2 groups; (b) for 3 groups; (c) for 4 groups. Highlighted cells indicate the samples assigned to each cluster (K) based on their highest posterior probability. Table S7: Permutational Multivariate Analysis of Variance results testing genetic differences between samples on a genetic distance matrix: (a) for the Occidental phylogeographic unit (Douro vs. other [file 12862_2025_2460_MOESM1_ESM.pdf]

Table S1: Details of the 115 samples used in this study, including the accession number from INSDC (when available – the others were retrieved from DRYAD), locality, phylogeographic unit, latitude, longitude, sex, and year of collection. For the Occidental phylogeographic unit samples collected in this study, additional information is provided on watershed of origin.

Sample	Accession number	Locality	Phylogeographic unit	Latitude	Longitude	Sex	Year of collection	Watershed	Data source reference
**SAMN18106615**	SAMN18106615	Hija de Dios	Central System	40.5	-4.9	Male	2019	-	[1]
**IBE-C1069**	-	Range Rio Calamantio	Iberian Range	42.2	-2.9	Female	<1997?	-	[2]
**IBE-C2736**	-	Rio Caranyo-Covelo	Occidental	42.3	-8.4	Female	<1997?	-	[2]
**IBE-C2737**	-	Rio Riobo-A Estrada	Occidental	42.7	-8.4	Male	<1997?	-	[2]
**IBE-C2739**	-	Rio Cabras-Laza	Occidental	42.1	-7.4	Male	<1997?	-	[2]
**IBE-C2740**	-	Rio Meladas-Carballeda	Occidental	42.2	-6.8	Female	<1997?	-	[2]
**IBE-C2749**	-	Rio Termes-As Neves	Occidental	42.1	-8.4	Female	<1997?	-	[2]
**IBE-C2755**	-	Leitzaran-Berastegi	Pyrenees	43.1	-1.9	Male	2001	-	[3]
**IBE-C2756**	-	Rio Aiaiturrieta-Ataun	Pyrenees	43.0	-2.1	Female	<1997?	-	[2]
**IBE-C2757**	-	Amundarain-Zaldibia	Pyrenees	43.1	-2.0	Female	<1997?	-	[2]
**IBE-C2758**	-	Amundarain-Zaldibia	Pyrenees	43.0	-2.1	Female	2006	-	[3]
**IBE-C2759**	-	Amundarain-Zaldibia	Pyrenees	43.0	-2.1	Male	2001	-	[3]
**IBE-C2760**	-	Erasote-Leitza	Pyrenees	43.1	-1.9	Female	2010	-	[3]
**IBE-C2761**	-	Erasote-Leitza	Pyrenees	43.1	-1.9	Female	2010	-	[3]
**IBE-C2762**	-	Erasote-Leitza	Pyrenees	43.1	-1.9	Male	2010	-	[3]

Sample	Accession number	Locality	Phylogeographic unit	Latitude	Longitude	Sex	Year of collection	Watershed	Data source reference
**IBE-C2763**	-	Olazar-Eugi	Pyrenees	43.0	-1.5	Male	2008	-	[3]
**IBE-C2765**	-	Ezpelura-Urrotz	Pyrenees	43.1	-1.7	Male	2010	-	[3]
**IBE-C2766**	-	Ezpelura-Urrotz	Pyrenees	43.1	-1.7	Female	1999	-	[3]
**SAMN18106612**	SAMN18106612	Ezpelura-Urrotz	Pyrenees	43.1	-1.7	Male	1999	-	[1]
**IBE-C2770**	-	Ezpelura-Urrotz	Pyrenees	43.1	-1.7	Female	1999	-	[3]
**IBE-C2771**	-	Ezpelura-Urrotz	Pyrenees	43.1	-1.7	Male	1999	-	[3]
**IBE-C2772**	-	Ezpelura-Urrotz	Pyrenees	43.1	-1.7	Male	1999	-	[3]
**IBE-C2773**	-	Ezpelura-Urrotz	Pyrenees	43.1	-1.7	Male	1999	-	[3]
**IBE-C2776**	-	Ezpelura-Urrotz	Pyrenees	43.1	-1.7	Female	1999	-	[3]
**IBE-C2778**	-	Ezpelura-Urrotz	Pyrenees	43.1	-1.7	Male	1999	-	[3]
**IBE-C2779**	-	Ezpelura-Urrotz	Pyrenees	43.1	-1.7	Female	1999	-	[3]
**IBE-C2780**	-	Ezpelura-Urrotz	Pyrenees	43.1	-1.7	Male	1999	-	[3]
**IBE-C2781**	-	Ameztia-Labaien	Pyrenees	43.1	-1.7	Female	1998	-	[3]
**IBE-C2782**	-	Ameztia-Labaien	Pyrenees	43.1	-1.7	Female	1998	-	[3]
**IBE-C2783**	-	Ameztia-Labaien	Pyrenees	43.1	-1.7	Female	1998	-	[3]
**IBE-C2784**	-	Ameztia-Labaien	Pyrenees	43.1	-1.7	Male	1998	-	[3]
**IBE-C2785**	-	Ameztia-Labaien	Pyrenees	43.1	-1.7	Female	1998	-	[3]
**IBE-C2786**	-	Ameztia-Labaien	Pyrenees	43.1	-1.7	Male	1998	-	[3]

Sample	Accession number	Locality	Phylogeographic unit	Latitude	Longitude	Sex	Year of collection	Watershed	Data source reference
**IBE-C2787**	-	Ameztia-Labaien	Pyrenees	43.1	-1.7	Male	1998	-	[3]
**IBE-C2788**	-	Ameztia-Labaien	Pyrenees	43.1	-1.7	Female	1998	-	[3]
**IBE-C2789**	-	Ameztia-Labaien	Pyrenees	43.1	-1.7	Female	1998	-	[3]
**IBE-C2790**	-	Ameztia-Labaien	Pyrenees	43.1	-1.7	Male	1998	-	[3]
**IBE-C2791**	-	Urrobi-Auritz	Pyrenees	43.0	-1.3	Male	2001	-	[3]
**IBE-C2792**	-	Urrobi-Auritz	Pyrenees	43.0	-1.3	Female	2001	-	[3]
**IBE-C2793**	-	Urrobi-Auritz	Pyrenees	43.0	-1.3	Female	2001	-	[3]
**IBE-C2794**	-	Urrobi-Auritz	Pyrenees	43.0	-1.3	Male	2001	-	[3]
**IBE-C2796**	-	Sasoaran-Eugi	Pyrenees	43.0	-1.6	Male	1997	-	[3]
**IBE-C2797**	-	Sasoaran-Eugi	Pyrenees	43.0	-1.6	Female	1997	-	[3]
**IBE-C2799**	-	Elama-Artikutza	Pyrenees	43.2	-1.8	Male	2002	-	[3]
**IBE-C2800**	-	Elama-Artikutza	Pyrenees	43.2	-1.8	Male	2002	-	[3]
**IBE-C2802**	-	Elama-Artikutza	Pyrenees	43.2	-1.8	Female	2002	-	[3]
**IBE-C2803**	-	Elama-Artikutza	Pyrenees	43.2	-1.8	Male	2002	-	[3]
**IBE-C2804**	-	Elama-Artikutza	Pyrenees	43.2	-1.8	Male	2002	-	[3]
**IBE-C2856**	-	Mount. Rio Lamedo	Cantabria	43.1	-4.6	Female	<1997?	-	[2]
**IBE-C2892**	-	Mount. Rio Cares-Cain	Cantabria	43.2	-4.9	Male	<1997?	-	[2]
**IBE-C2894**	-	Mount. Rio Cares-Casiellos	Cantabria	43.2	-4.9	Female	<1997?	-	[2]

Sample	Accession number	Locality	Phylogeographic unit	Latitude	Longitude	Sex	Year of collection	Watershed	Data source reference
**IBE-C3301**	-	Paiva-Fraguas	Occidental	40.8	-7.8	Female	<1997?	-	[2]
**IBE-C3303**	-	Sabor-Macas-Quintanilla	Occidental	41.8	-6.6	Male	<1997?	-	[2]
**IBE-C3305**	-	Paiva-Fraguas	Occidental	40.8	-7.8	Male	<1997?	-	[2]
**IBE-C3733**	-	Oja Cabecera	Iberian Range	42.2	-3.1	Male	2011	-	[4]
**SAMN18106613**	SAMN18106613	Oja Cabecera	Iberian Range	42.2	-3.1	Female	2011	-	[1]
**IBE-C3735**	-	Oja Cabecera	Iberian Range	42.2	-3.1	Female	2011	-	[4]
**IBE-C3736**	-	Oja Cabecera	Iberian Range	42.2	-3.1	Female	2011	-	[4]
**IBE-C3737**	-	Oja Cabecera	Iberian Range	42.2	-3.1	Female	2011	-	[4]
**IBE-C3738**	-	Oja Azarrulla	Iberian Range	42.3	-3.0	Female	2011	-	[4]
**IBE-C3739**	-	Ciloria	Iberian Range	42.3	-3.1	Female	2011	-	[4]
**IBE-C3740**	-	Ciloria	Iberian Range	42.3	-3.1	Male	2011	-	[4]
**IBE-C3741**	-	Tobía	Iberian Range	42.3	-2.9	Female	2011	-	[4]
**IBE-C3742**	-	Tobía	Iberian Range	42.2	-2.9	Male	2011	-	[4]
**IBE-C3743**	-	Tobía	Iberian Range	42.3	-2.9	Male	2011	-	[4]
**IBE-C3744**	-	Tobía	Iberian Range	42.2	-2.9	Male	2011	-	[4]
**IBE-C3745**	-	Cárdenas	Iberian Range	42.3	-2.9	Female	2011	-	[4]
**IBE-C3746**	-	Roñas	Iberian Range	42.2	-2.8	Male	2011	-	[4]
**IBE-C3747**	-	Roñas	Iberian Range	42.2	-2.8	Female	2011	-	[4]

Sample	Accession number	Locality	Phylogeographic unit	Latitude	Longitude	Sex	Year of collection	Watershed	Data source reference
**IBE-C3748**	-	Roñas	Iberian Range	42.2	-2.8	Male	2011	-	[4]
**IBE-C3749**	-	Roñas	Iberian Range	42.2	-2.8	Female	2011	-	[4]
**IBE-C3750**	-	Range Iregua	Iberian Range	42.1	-2.7	Male	2011	-	[2]
**IBE-C3751**	-	Iregua-Cabecera	Iberian Range	42.1	-2.7	Male	2011	-	[4]
**IBE-C3752**	-	La Vieja	Iberian Range	42.1	-2.6	Male	2011	-	[4]
**IBE-C3753**	-	La Vieja	Iberian Range	42.0	-2.6	Male	2011	-	[4]
**IBE-C3754**	-	Range La Vieja	Iberian Range	42.0	-2.6	Female	2011	-	[2]
**IBE-C3755**	-	La Vieja	Iberian Range	42.1	-2.6	Female	2011	-	[4]
**IBE-C3756**	-	Range La Soledad	Iberian Range	42.2	-3.1	Male	<1997?	-	[2]
**IBE-C3757**	-	La Soledad	Iberian Range	42.2	-3.1	Female	2011	-	[4]
**IBE-C3758**	-	La Soledad	Iberian Range	42.2	-3.1	Female	2011	-	[4]
**IBE-C3759**	-	Urumea	Pyrenees	43.1	-1.8	Female	2011	-	[3]
**IBE-C3760**	-	Urumea	Pyrenees	43.1	-1.8	Male	2011	-	[3]
**IBE-C3761**	-	Urumea	Pyrenees	43.1	-1.8	Female	2011	-	[3]
**IBE-C3763**	-	Aritzakun	Pyrenees	43.3	-1.4	Male	2011	-	[3]
**IBE-C3765**	-	Urdanta	Iberian Range	42.3	-3.0	Female	2011	-	[4]
**IBE-C3766**	-	Urdanta	Iberian Range	42.3	-3.0	Female	2011	-	[4]
**IBE-C3767**	-	Urdanta	Iberian Range	42.3	-3.0	Male	2011	-	[4]

Sample	Accession number	Locality	Phylogeographic unit	Latitude	Longitude	Sex	Year of collection	Watershed	Data source reference
**IBE-C3768**	-	Urdanta	Iberian Range	42.3	-3.0	Female	2011	-	[4]
**IBE-C3769**	-	Urdanta	Iberian Range	42.3	-3.0	Male	2011	-	[4]
**IBE-C3770**	-	Urdanta	Iberian Range	42.3	-3.0	Female	2011	-	[4]
**IBE-C3771**	-	Ormázal	Iberian Range	42.1	-2.8	Male	2011	-	[4]
**IBE-C3772**	-	Mayor	Iberian Range	42.1	-2.7	Male	2011	-	[4]
**SAMN18106614**	SAMN18106614	Mayor	Iberian Range	42.1	-2.7	Male	2011	-	[1]
**IBE-C3774**	-	Iregua Achichuelo	Iberian Range	42.1	-2.7	Female	2011	-	[4]
**IBE-C3775**	-	Iregua Achichuelo	Iberian Range	42.1	-2.7	Female	2011	-	[4]
**IBE-C422**	-	Tor	Pyrenees	42.6	1.4	Female	<1997?	-	[2]
**IBE-C485**	-	System Rio Ambroz-Hervas	Central System	40.3	-5.8	Female	<1997?	-	[2]
**SAMN18106611**	SAMN18106611	Torán	Pyrenees	42.8	-0.7	Male	2017	-	[1]
**SAMN18106616**	SAMN18106616	Requejo	Occidental	42.03	-6.7	Male	2018	-	[1]
**IBE-S1925**	-	Riu Ritort-Camprodon	Pyrenees	42.3	2.4	Male	<1997?	-	[2]
**1**	SAMEA118002938	Espadanedo	Occidental	41.65158321	-6.933384732	Male	2015	Tua	This study
**2**	SAMEA118002939	Felgar	Occidental	41.22312188	-6.947056639	Female	2015	Sabor	This study
**3**	SAMEA118002940	Quintela de Lampaças	Occidental	41.63340474	-6.866797096	Female	2015	Sabor	This study
**4**	SAMEA118002941	Chacim	Occidental	41.46954975	-6.87620478	Female	2016	Sabor	This study
**5**	SAMEA118002942	Gebelim	Occidental	41.43624515	-6.91739072	Female	2016	Sabor	This study

Sample	Accession number	Locality	Phylogeographic unit	Latitude	Longitude	Sex	Year of collection	Watershed	Data source reference
**6**	SAMEA118002943	Coelhoso	Occidental	41.665592	-6.658344	Female	2016	Sabor	This study
**7**	SAMEA118002944	Rebordãos	Occidental	41.739392	-6.779879	Female	2016	Sabor	This study
**8**	SAMEA118002945	Serapicos	Occidental	41.647539	-6.724812	Female	2016	Sabor	This study
**9**	SAMEA118002946	Edrosa	Occidental	41.730551	-6.935536	Female	2016	Tua	This study
**10**	SAMEA118002947	Montouto	Occidental	41.92936672	-6.97219392	Female	2016	Tua	This study
**11**	SAMEA118002948	Soutelo	Occidental	41.90403	-6.796208333	Female	2016	Sabor	This study
**12**	SAMEA118002949	Outeiro	Occidental	42.531308	-7.117328	Male	2012	Minho	This study
**13**	SAMEA118002950	Ferramulín	Occidental	42.57475	-7.0592	Female	2012	Minho	This study
**14**	SAMEA118002951	Riobó	Occidental	42.735563	-8.401418	Male	2013	Ulla	This study
**15**	SAMEA118002952	Riobó	Occidental	42.735563	-8.401418	Male	2013	Ulla	This study

Table S2: Details of the 115 samples used in this study, including the total number of raw reads, mapping rate (as a percentage), duplication rate (as a percentage) and mean coverage for each sample before filtering, and number of SNPs, mean depth and the proportion of sites that are missing in a sample after *bcftools* (BT) and *vcftools* (VT) filtering steps.

Sample	Total number of raw reads	Mapping rate	Duplication rate	Mean coverage	Number of SNPs (BT)	Mean depth (BT)	Missing sites (BT)	Number of SNPs (VT)	Mean depth (VT)	Missing sites (VT)
IBE-C1069	2034450	90.86	33.44	0.1299	131166328	0.144122	0.944165	110	13.4182	0.00909091
IBE-C2736	1011797	87.97	54.39	0.0653	131166328	0.0714311	0.990353	110	13.6182	0.118182
IBE-C2737	1924394	90.92	55.94	0.1281	131166328	0.145278	0.990346	110	20.3182	0.0272727
IBE-C2739	2637335	93.35	60.29	0.1821	131166328	0.203152	0.992662	110	42.8455	0.0909091
IBE-C2740	2775531	24.12	31.48	0.0459	131166328	0.0504522	0.9823	110	8.39091	0.145455
IBE-C2749	2084163	24.35	50.6	0.0367	131166328	0.0391358	0.992375	110	9.30909	0.0818182
IBE-C2755	2055750	93.97	60.11	0.1421	131166328	0.142296	0.994309	110	31.3909	0.109091
IBE-C2756	4936586	91.68	37.53	0.3291	131166328	0.373514	0.977329	110	48.6091	0.0363636
IBE-C2757	3886952	86.46	46.33	0.2473	131166328	0.28734	0.987215	110	60	0.00909091
IBE-C2758	2458337	92.21	58.73	0.1652	131166328	0.177689	0.992393	110	34.6182	0.0545455
IBE-C2759	4980296	91.44	49.85	0.3312	131166328	0.350162	0.984773	110	56.9545	0.0727273
IBE-C2760	2960005	91.04	59.92	0.1962	131166328	0.221326	0.993023	110	30.8364	0.154545
IBE-C2761	5961091	93.1	53.22	0.4029	131166328	0.392951	0.980709	110	52.7727	0.0545455
IBE-C2762	8093377	92.45	49.6	0.5464	131166328	0.596484	0.987564	110	105.427	0
IBE-C2763	3002097	91.25	54.34	0.1995	131166328	0.218273	0.990064	110	36.6636	0.0454545
IBE-C2765	1026994	90.63	45.34	0.0679	131166328	0.0726334	0.988863	110	16.9273	0.0454545
IBE-C2766	4224186	90.86	48.56	0.2785	131166328	0.300865	0.986791	110	43.5909	0.0272727
IBE-C2770	6297156	91.75	55.23	0.422	131166328	0.450103	0.99041	110	73.5364	0.0454545
IBE-C2771	5511207	93.45	54.45	0.3791	131166328	0.383102	0.991206	110	82.1364	0.0636364

Sample	Total number of raw reads	Mapping rate	Duplication rate	Mean coverage	Number of SNPs (BT)	Mean depth (BT)	Missing sites (BT)	Number of SNPs (VT)	Mean depth (VT)	Missing sites (VT)
IBE-C2772	2675478	91.98	60.29	0.1801	131166328	0.189228	0.992256	110	34.8909	0.0272727
IBE-C2773	2706390	91.7	54.44	0.1809	131166328	0.1993	0.990375	110	35.4364	0.0272727
IBE-C2776	3744171	90.45	53.89	0.2462	131166328	0.276981	0.991129	110	38.4364	0.0727273
IBE-C2778	3315499	93.3	59.41	0.2268	131166328	0.234974	0.993858	110	44.3545	0.1
IBE-C2779	2884397	94.13	57.6	0.2	131166328	0.202032	0.994001	110	47.1273	0.109091
IBE-C2780	1477552	93.54	60.23	0.1015	131166328	0.104041	0.994727	110	20.7182	0.227273
IBE-C2781	3343999	91.03	49.47	0.2211	131166328	0.242782	0.988966	110	40.8455	0.0272727
IBE-C2782	2289454	93.64	58.64	0.1574	131166328	0.162459	0.993944	110	33.0364	0.109091
IBE-C2783	4655418	93.24	52.86	0.3189	131166328	0.326728	0.989841	110	67.8727	0.0545455
IBE-C2784	2332204	91.1	55.17	0.1548	131166328	0.165748	0.990314	110	24.8636	0.0909091
IBE-C2785	5662179	91.92	50.94	0.3794	131166328	0.410649	0.987866	110	66.9545	0
IBE-C2786	2803468	90.55	60.82	0.1846	131166328	0.198843	0.994286	110	34.3182	0.209091
IBE-C2787	9257109	90.86	54.58	0.6118	131166328	0.651196	0.991489	110	107.773	0.0545455
IBE-C2788	8677240	91.03	55.03	0.5731	131166328	0.614848	0.992567	110	94.8273	0.190909
IBE-C2789	14451258	91.06	51	0.9572	131166328	1.02051	0.988391	110	172.455	0.0181818
IBE-C2790	5543162	92.52	51.52	0.3746	131166328	0.406416	0.989467	110	78.3182	0.0181818
IBE-C2791	3912245	91.2	52.09	0.2593	131166328	0.28736	0.989559	110	45.3545	0.0181818
IBE-C2792	5914177	91.05	57.58	0.3912	131166328	0.431426	0.992427	110	65.8091	0.127273
IBE-C2793	6226362	90.73	43.07	0.4095	131166328	0.457878	0.985399	110	73.0364	0
IBE-C2794	1239874	90.52	56.88	0.0817	131166328	0.089393	0.992931	110	13.9636	0.136364
IBE-C2796	3263897	91.98	47.9	0.2186	131166328	0.232768	0.987917	110	43.3545	0.00909091
IBE-C2797	2395890	91.32	49.27	0.1589	131166328	0.171603	0.989446	110	29.9273	0.0272727

Sample	Total number of raw reads	Mapping rate	Duplication rate	Mean coverage	Number of SNPs (BT)	Mean depth (BT)	Missing sites (BT)	Number of SNPs (VT)	Mean depth (VT)	Missing sites (VT)
IBE-C2799	1982105	91.93	59.77	0.1334	131166328	0.14324	0.992491	110	24.7182	0.0909091
IBE-C2800	1307909	89.92	57.51	0.0857	131166328	0.0900856	0.993606	110	18.4727	0.136364
IBE-C2802	2267684	90.66	46.37	0.1497	131166328	0.15665	0.988955	110	32.5636	0.0181818
IBE-C2803	3033378	91.01	53.83	0.2007	131166328	0.213689	0.992011	110	39.3818	0.0454545
IBE-C2804	4668208	92.47	58.47	0.3152	131166328	0.335887	0.993519	110	64.2091	0.127273
IBE-C2856	2214155	90.95	58.08	0.1476	131166328	0.166041	0.991486	110	26.8	0.0636364
IBE-C2892	203878	86.2	49.93	0.0128	131166328	0.0146361	0.994956	110	2.25455	0.336364
IBE-C2894	248379	87.72	35.43	0.0157	131166328	0.0177477	0.992434	110	2.58182	0.309091
IBE-C3301	2612432	87.73	41.04	0.169	131166328	0.186751	0.984301	110	40.8636	0.0272727
IBE-C3303	854352	88.25	27.88	0.0534	131166328	0.0570526	0.974669	110	11.5091	0.0909091
IBE-C3305	722376	87.45	26.3	0.0463	131166328	0.0502097	0.98277	110	14.0909	0.0636364
IBE-C3733	4333991	87.22	57.42	0.2746	131166328	0.298846	0.987301	110	48.2273	0.0818182
IBE-C3735	3014159	89.87	59.24	0.1952	131166328	0.210379	0.980925	110	29.3818	0.127273
IBE-C3736	2180584	79.55	63.72	0.1268	131166328	0.149667	0.991949	110	23.8727	0.127273
IBE-C3737	7959967	83.99	56.23	0.4896	131166328	0.5804	0.98732	110	95.7182	0
IBE-C3738	1827852	88.44	61.09	0.1178	131166328	0.133848	0.991034	110	19.3909	0.209091
IBE-C3739	3630939	89.02	61.84	0.2381	131166328	0.260357	0.991545	110	65.3545	0.0363636
IBE-C3740	3470264	90.48	59.47	0.2297	131166328	0.252941	0.989668	110	46.9909	0.00909091
IBE-C3741	3863617	86.51	58.77	0.2421	131166328	0.261645	0.990291	110	36.6818	0.145455
IBE-C3742	3546597	81.04	60.34	0.2088	131166328	0.228026	0.990009	110	36.8727	0.0727273
IBE-C3743	3102519	89.57	60.95	0.2048	131166328	0.230184	0.99177	110	55.3455	0.0545455
IBE-C3744	2795163	85.16	62.5	0.1745	131166328	0.207549	0.991076	110	38.3091	0.0545455

Sample	Total number of raw reads	Mapping rate	Duplication rate	Mean coverage	Number of SNPs (BT)	Mean depth (BT)	Missing sites (BT)	Number of SNPs (VT)	Mean depth (VT)	Missing sites (VT)
IBE-C3745	1476004	86.5	62.35	0.0933	131166328	0.106776	0.992156	110	15.9545	0.145455
IBE-C3746	2169694	88.02	58.91	0.1381	131166328	0.149889	0.989207	110	23.6455	0.154545
IBE-C3747	5873897	88.87	59.16	0.384	131166328	0.451603	0.989479	110	82.3455	0.0454545
IBE-C3748	1957770	89.66	56.54	0.1282	131166328	0.148094	0.990204	110	26.9909	0.118182
IBE-C3749	3253756	92.54	62.45	0.2211	131166328	0.242564	0.989865	110	49.7273	0.0181818
IBE-C3750	1813793	88.81	55.94	0.1183	131166328	0.134218	0.990388	110	23.3818	0.0636364
IBE-C3751	11062283	87.18	59.73	0.7004	131166328	0.800273	0.987126	110	113.645	0.0363636
IBE-C3752	5820592	88.55	58.16	0.3784	131166328	0.439585	0.988747	110	99.7273	0
IBE-C3753	6002916	77.29	54.15	0.3359	131166328	0.369699	0.984008	110	57.2909	0.0454545
IBE-C3754	7195244	84.85	32.37	0.4462	131166328	0.50605	0.975749	110	85.3455	0.00909091
IBE-C3755	8055321	88.54	60.15	0.5177	131166328	0.569491	0.988185	110	78.0273	0.1
IBE-C3756	5569205	90.7	45.52	0.3702	131166328	0.403388	0.982807	110	66.5364	0.00909091
IBE-C3757	7338254	90.55	59.62	0.4833	131166328	0.518197	0.990062	110	104.927	0.0818182
IBE-C3758	2507142	90.91	62.7	0.1656	131166328	0.179101	0.991285	110	30.2091	0.227273
IBE-C3759	2183830	88.85	60.18	0.1413	131166328	0.15281	0.993693	110	24.6818	0.172727
IBE-C3760	6340281	91.25	47.79	0.4203	131166328	0.464353	0.988063	110	77.8364	0
IBE-C3761	3608024	91.02	58.53	0.2384	131166328	0.257681	0.993048	110	35.9727	0.127273
IBE-C3763	5116317	92.07	59.84	0.3444	131166328	0.375589	0.993045	110	72.4182	0.0636364
IBE-C3765	2316150	81.13	56.78	0.1377	131166328	0.158537	0.990302	110	28.1636	0.0818182
IBE-C3766	11704257	15.26	55.4	0.1286	131166328	0.144339	0.99069	110	31.9182	0.0363636
IBE-C3767	5423159	67.76	51.04	0.2661	131166328	0.295905	0.970998	110	36.5909	0.0363636
IBE-C3768	12215261	84.94	49.89	0.7509	131166328	0.835774	0.977126	110	117.127	0.0636364

Sample	Total number of raw reads	Mapping rate	Duplication rate	Mean coverage	Number of SNPs (BT)	Mean depth (BT)	Missing sites (BT)	Number of SNPs (VT)	Mean depth (VT)	Missing sites (VT)
IBE-C3769	4351626	72.07	63.23	0.2258	131166328	0.247115	0.989282	110	41.7727	0.145455
IBE-C3770	1500452	78.43	63.14	0.0861	131166328	0.10129	0.992874	110	16.7091	0.181818
IBE-C3771	10280980	85.77	52.31	0.6404	131166328	0.708156	0.984016	110	121.009	0.0363636
IBE-C3772	5666969	87.44	58.62	0.3602	131166328	0.379638	0.99058	110	75.1909	0.0909091
IBE-C3774	11869363	84.42	47.41	0.7286	131166328	0.837553	0.979679	110	150.4	0
IBE-C3775	6795664	36.84	36.74	0.1784	131166328	0.18949	0.979029	110	36.5818	0
IBE-C422	2982868	93.71	56.35	0.2064	131166328	0.224162	0.991981	110	42.6727	0.118182
IBE-C485	1540134	90.11	68.19	0.1026	131166328	0.115986	0.992816	110	24.4545	0.0909091
IBE-S1925	1285251	91.78	55.53	0.0871	131166328	0.0986456	0.992358	110	19.8455	0.145455
SAMN18106611	2430364536	86.21	38.94	99.427	131166328	98.7452	1.05667e-05	110	671.136	0
SAMN18106612	133311010	88.3	7.12	7.5254	131166328	7.76101	0.00377	110	98.0091	0
SAMN18106613	164266428	87.72	7.03	9.3762	131166328	9.97958	0.002137	110	140.109	0
SAMN18106614	180798280	85.97	7.3	10.1449	131166328	10.5815	0.001863	110	166.591	0
SAMN18106615	423518938	87.6	11.23	24.5675	131166328	25.8816	0.00103	110	404.082	0
SAMN18106616	186336338	88.18	7.73	10.7286	131166328	11.323	0.001675	110	197.336	0
1	2571490	95.22	61.06	0.1194	131166328	0.105069	0.993311	110	27.5091	0
2	1586050	95.65	60.21	0.0734	131166328	0.101765	0.992953	110	20.4364	0.00909091
3	2032396	95.69	61.73	0.0948	131166328	0.142602	0.992988	110	39.7182	0
4	3133188	96.31	61.63	0.1544	131166328	0.0934875	0.993572	110	22.1909	0
5	3002750	96.05	61.9	0.1485	131166328	0.116344	0.993439	110	27.8727	0
6	2912628	95.97	60.77	0.1423	131166328	0.0718746	0.994029	110	15.8909	0.0454545
7	2579686	96.3	60.72	0.1267	131166328	0.0934832	0.993614	110	20.9909	0

Sample	Total number of raw reads	Mapping rate	Duplication rate	Mean coverage	Number of SNPs (BT)	Mean depth (BT)	Missing sites (BT)	Number of SNPs (VT)	Mean depth (VT)	Missing sites (VT)
8	3076754	96.1	62.36	0.151	131166328	0.149222	0.992904	110	42.0727	0
9	1329370	95.94	59.36	0.0652	131166328	0.145654	0.993104	110	40.2	0
10	2292568	96.12	60.5	0.1157	131166328	0.138682	0.993059	110	35.9636	0
11	548750	96.58	49.79	0.0298	131166328	0.121789	0.993201	110	27.4091	0
12	2407438	95.55	59.56	0.1096	131166328	0.145688	0.993012	110	30.7909	0.00909091
13	2308712	95.49	58.91	0.1039	131166328	0.0639595	0.994055	110	14.1091	0.0454545
14	2968928	95.84	61.21	0.1452	131166328	0.111035	0.993196	110	31.0727	0
15	2082848	95.95	59.8	0.0977	131166328	0.0280127	0.995159	110	8.53636	0.109091

Table S3: Details of the samples used in this study from the Occidental phylogeographic unit, including the number of SNPs, mean depth and the proportion of sites that are missing in a sample after *bcftools* (BT) and *vcftools* (VT) filtering steps, for all the 15, 14 (Occidental), 11 (Douro) and 8 (Sabor) samples.

Sample	Number of SNPs (BT)	Mean depth (BT)	Missing sites (BT)	Number of SNPs (VT)	Mean depth (VT)	Missing sites (VT)	Number of SNPs (VT) Occidental	Mean depth (VT) Occidental	Missing sites (VT) Occidental	Number of SNPs (VT) Douro	Mean depth (VT) Douro	Missing sites (VT) Douro	Number of SNPs (VT) Sabor	Mean depth (VT) Sabor	Missing sites (VT) Sabor
1	131166328	0.116344	0.993439	7711	23.9283	0.005187	7604	23.6774	0.006049	8771	19.5886	0.013567			
2	131166328	0.0718746	0.994029	7711	14.913	0.015562	7604	14.7774	0.017096	8771	12.2324	0.040474	8541	14.3041	0
3	131166328	0.0934832	0.993614	7711	19.2416	0.007911	7604	19.0592	0.009074	8771	15.8127	0.01847	8541	18.5907	0
4	131166328	0.149222	0.992904	7711	30.9087	0.001427	7604	30.6977	0.001841	8771	25.058	0.004903	8541	29.9379	0
5	131166328	0.145654	0.993104	7711	30.3083	0.001556	7604	30.0504	0.00171	8771	24.6887	0.005359	8541	29.4866	0
6	131166328	0.138682	0.993059	7711	28.5985	0.002334	7604	28.3812	0.002499	8771	23.3729	0.004789	8541	27.7106	0
7	131166328	0.121789	0.993201	7711	25.1752	0.003112	7604	24.9759	0.003419	8771	20.5694	0.009463	8541	24.4422	0
8	131166328	0.145688	0.993012	7711	29.693	0.002983	7604	29.441	0.004208	8771	24.2687	0.008551	8541	28.788	0
9	131166328	0.0639595	0.994055	7711	13.2565	0.015692	7604	13.1432	0.017359	8771	10.8316	0.041272			
10	131166328	0.111035	0.993196	7711	22.665	0.004669	7604	22.5384	0.004603	8771	18.5297	0.011059			
11	131166328	0.0280127	0.995159	7711	5.71768	0.151083	7604	5.7091	0.151499	8771	4.54019	0.276137	8541	6.05959	0
12	131166328	0.105069	0.993311	7711	21.5113	0.011412	7604	21.2897	0.012756						
13	131166328	0.101765	0.992953	7711	20.6882	0.010115	7604	20.4879	0.010652						
14	131166328	0.142602	0.992988	7711	29.4775	0.002594	7604	29.2029	0.003156						
15	131166328	0.0934875	0.993572	7711	19.3448	0.008689									

Table S4: Genetic distances between individuals (see text for further details): **a.** for the Occidental phylogeographic unit; **b.** for the Douro river system; **c.** for the Sabor watershed.

a

**Sample**	**1**	**2**	**3**	**4**	**5**	**6**	**7**	**8**	**9**	**10**	**11**	**12**	**13**	**14**
**1**	0.0000													
**2**	0.2772	0.0000												
**3**	0.2569	0.2683	0.0000											
**4**	0.2792	0.2490	0.2561	0.0000										
**5**	0.2894	0.2484	0.2800	0.2726	0.0000									
**6**	0.2710	0.2597	0.2719	0.2720	0.2714	0.0000								
**7**	0.2677	0.2764	0.2783	0.2779	0.2885	0.2247	0.0000							
**8**	0.2803	0.2662	0.2786	0.2649	0.2836	0.2717	0.2766	0.0000						
**9**	0.2338	0.2760	0.2654	0.2786	0.2873	0.2745	0.2709	0.2778	0.0000					
**10**	0.2514	0.2783	0.2784	0.2914	0.2954	0.2793	0.2860	0.2956	0.2647	0.0000				
**11**	0.2571	0.2699	0.2697	0.2751	0.2853	0.2661	0.2685	0.2699	0.2595	0.2645	0.0000			
**12**	0.2863	0.2946	0.2955	0.3000	0.3074	0.2962	0.2992	0.3048	0.2856	0.2805	0.2750	0.0000		
**13**	0.2854	0.2978	0.3005	0.3049	0.3091	0.3017	0.2996	0.3063	0.2889	0.2813	0.2859	0.2583	0.0000	
**14**	0.2957	0.3017	0.3035	0.3134	0.3147	0.3013	0.3155	0.3196	0.3010	0.2906	0.2881	0.2850	0.2951	0.0000

b

Sample	1	2	3	4	5	6	7	8	9	10	11
**1**	0.0000										
**2**	0.2559	0.0000									
**3**	0.2383	0.2470	0.0000								
**4**	0.2594	0.2337	0.2375	0.0000							
**5**	0.2673	0.2309	0.2577	0.2543	0.0000						
**6**	0.2504	0.2390	0.2496	0.2515	0.2498	0.0000					
**7**	0.2472	0.2532	0.2545	0.2577	0.2650	0.2122	0.0000				
**8**	0.2557	0.2429	0.2522	0.2433	0.2602	0.2464	0.2533	0.0000			
**9**	0.2209	0.2556	0.2451	0.2576	0.2663	0.2546	0.2505	0.2552	0.0000		
**10**	0.2373	0.2568	0.2562	0.2696	0.2744	0.2588	0.2647	0.2684	0.2471	0.0000	
**11**	0.2374	0.2464	0.2480	0.2515	0.2654	0.2449	0.2488	0.2465	0.2380	0.2414	0.0000

c

Sample	1	2	3	4	5	6	7	8
**1**	0.0000							
**2**	0.2361	0.0000						
**3**	0.2180	0.2179	0.0000					
**4**	0.2166	0.2336	0.2235	0.0000				
**5**	0.2257	0.2262	0.2253	0.2219	0.0000			
**6**	0.2419	0.2379	0.2321	0.2374	0.1966	0.0000		
**7**	0.2320	0.2370	0.2217	0.2334	0.2219	0.2350	0.0000	
**8**	0.2590	0.2547	0.2523	0.2601	0.2466	0.2537	0.2521	0.0000

Table S5: Matrix of overland (dark green) and/or river (dark blue) distances between samples, in meters: **a.** for the Occidental phylogeographic unit; **b.** for the Douro river system; **c.** for the Sabor watershed.

**a**	**Overland distances**													
**Samples**	**1**	**2**	**3**	**4**	**5**	**6**	**7**	**8**	**9**	**10**	**11**	**12**	**13**	**14**
**1**	0.00	47709.74	5897.46	20816.07	24008.33	22927.92	16073.40	17355.03	8792.47	31090.03	30321.85	99102.28	103290.29	170933.01
**2**		0.00	46161.13	28063.98	23854.03	54831.60	59137.87	50756.18	56494.92	78646.68	76832.59	146308.78	150749.15	206949.69
**3**			0.00	18257.08	22348.95	17705.60	13835.28	11916.39	12231.44	34088.20	30690.65	102074.65	105988.00	176280.73
**4**				0.00	5055.04	28381.17	31090.39	23486.40	29471.33	51804.62	48821.18	119865.60	123957.47	189018.81
**5**					0.00	33429.64	35635.48	28472.83	32796.80	55082.80	53039.58	123019.29	127279.42	189603.10
**6**						0.00	13020.11	5882.19	24147.74	39250.48	28904.56	103559.42	106481.46	186671.15
**7**							0.00	11202.84	12967.85	26488.92	18377.41	92451.97	95805.63	173653.46
**8**								0.00	19805.35	37494.78	29161.16	103586.31	106846.50	183817.28
**9**									0.00	22339.96	22506.68	90394.38	94528.31	164659.92
**10**										0.00	14848.06	68067.25	72200.54	147940.50
**11**											0.00	74678.22	77746.40	161318.27
**12**												0.00	6790.28	107593.15
**13**													0.00	111333.92
**14**														0.00

b

Samples	1	2	3	4	5	6	7	8	9	10	11
						Overland distances					
1	0.00	47709.74	5897.46	20816.07	24008.33	22927.92	16073.40	17355.03	8792.47	31090.03	30321.85
2	165752.94	0.00	46161.13	28063.98	23854.03	54831.60	59137.87	50756.18	56494.92	78646.68	76832.59
3	235678.37	73962.62	0.00	18257.08	22348.95	17705.60	13835.28	11916.39	12231.44	34088.20	30690.65
4	212054.38	50338.64	28516.99	0.00	5055.04	28381.17	31090.39	23486.40	29471.33	51804.62	48821.18
5	200201.37	38485.62	94213.50	70589.52	0.00	33429.64	35635.48	28472.83	32796.80	55082.80	53039.58
6	239176.61	77460.86	84150.52	60526.54	97711.74	0.00	13020.11	5882.19	24147.74	39250.48	28904.56
7	260177.40	98461.65	105151.30	81527.32	118712.53	21000.78	0.00	11202.84	12967.85	26488.92	18377.41
8	233146.31	71430.57	78120.22	54496.24	91681.45	65302.24	86303.02	0.00	19805.35	37494.78	29161.16
9	40056.64	166913.14	236838.57	213214.59	201361.58	240336.82	261337.60	234306.52	0.00	22339.96	22506.68
10	83561.88	191051.96	260977.39	237353.41	225500.40	264475.64	285476.42	258445.34	84722.08	0.00	14848.06
11	283503.66	121787.91	128477.56	104853.58	142038.79	44327.05	45277.17	109629.29	284663.86	308802.69	0.00
						River distances					

c

Samples	2	3	4	5	6	7	8	11
				Overland distances				
2	0.00	46161.13	28063.98	23854.03	54831.60	59137.87	50756.18	76832.59
3	73962.62	0.00	18257.08	22348.95	17705.60	13835.28	11916.39	30690.65
4	50338.64	28516.99	0.00	5055.04	28381.17	31090.39	23486.40	48821.18
5	38485.62	94213.50	70589.52	0.00	33429.64	35635.48	28472.83	53039.58
6	77460.86	84150.52	60526.54	97711.74	0.00	13020.11	5882.19	28904.56
7	98461.65	105151.30	81527.32	118712.53	21000.78	0.00	11202.84	18377.41
8	71430.57	78120.22	54496.24	91681.45	65302.24	86303.02	0.00	29161.16
11	121787.91	128477.56	104853.58	142038.79	44327.05	45277.17	109629.29	0.00
				River distances				

Table S6: Discriminant Analysis of Principal Components posterior probabilities for the Occidental phylogeographic unit, taking 6 principal components into account: **a.** for 2 groups; **b.** for 3 groups; **c.** for 4 groups. Highlighted cells indicate the samples assigned to each cluster (K) based on their highest posterior probability.

**a**	**Group (K)**	
**Sample**	**1**	**2**
**1**	1.00E+00	3.60E-31
**2**	1.00E+00	3.34E-26
**3**	1.00E+00	1.74E-29
**4**	1.00E+00	4.54E-25
**5**	1.00E+00	4.24E-30
**6**	1.00E+00	1.84E-27
**7**	1.00E+00	2.97E-29
**8**	1.00E+00	1.07E-29
**9**	1.00E+00	3.76E-34
**10**	1.00E+00	3.75E-18
**11**	1.00E+00	5.38E-18
**12**	6.40E-24	1.00E+00
**13**	3.17E-28	1.00E+00
**14**	6.06E-26	1.00E+00

**b**		**Group (K)**	
**Sample**	**1**	**2**	**3**
**1**	1.00E+00	7.91E-29	1.46E-29
**2**	4.04E-21	8.89E-48	1.00E+00
**3**	2.68E-27	2.35E-57	1.00E+00
**4**	4.11E-37	9.68E-64	1.00E+00
**5**	2.46E-34	2.13E-65	1.00E+00
**6**	1.00E+00	8.59E-26	8.38E-24
**7**	1.00E+00	1.36E-26	3.85E-37
**8**	1.00E+00	2.55E-27	4.31E-32
**9**	1.00E+00	1.48E-31	1.18E-29
**10**	1.00E+00	1.18E-16	2.28E-33
**11**	1.00E+00	7.04E-17	1.50E-27
**12**	6.26E-22	1.00E+00	6.01E-53
**13**	3.26E-26	1.00E+00	1.41E-62
**14**	4.65E-24	1.00E+00	1.99E-59

c	Group (K)			
**Samples**	**1**	**2**	**3**	**4**
**1**	1.00E+00	1.77E-64	9.40E-15	1.72E-34
**2**	3.80E-11	9.22E-91	1.00E+00	2.16E-44
**3**	1.00E+00	3.59E-67	1.80E-08	1.40E-34
**4**	2.30E-08	2.30E-87	1.00E+00	7.46E-39
**5**	2.17E-15	8.32E-104	1.00E+00	2.17E-51
**6**	9.42E-13	9.06E-91	1.00E+00	2.04E-49
**7**	4.59E-13	9.78E-102	1.00E+00	1.78E-46
**8**	7.88E-14	1.50E-99	1.00E+00	7.50E-50
**9**	1.00E+00	1.41E-68	2.29E-13	6.38E-37
**10**	1.00E+00	2.19E-52	5.44E-17	5.39E-23
**11**	1.00E+00	7.45E-55	9.46E-08	6.66E-27
**12**	1.39E-23	2.95E-61	7.51E-41	1.00E+00
**13**	3.53E-37	5.87E-94	1.06E-50	1.00E+00
**14**	4.27E-60	1.00E+00	4.15E-94	5.26E-77

Table S7: Permutational Multivariate Analysis of Variance results testing genetic differences between samples on a genetic distance matrix: **a.** for the Occidental phylogeographic unit (Douro vs other river systems); **b.** for the Douro river system (Tua vs Sabor watersheds). Df stands for degrees of freedom, SS for sum of squares, R^2 for proportion of variation explained and F for F-statistic. Values with $p < 0.01$ are highlighted in bold.

**a**	**Df**	**SS**	**R^2**	**F**	**p-value**
**Model**	1	0.0688	0.1332	1.8447	**0.005**
**Residual**	12	0.4476	0.8668		
**Total**	13	0.5164	1.0000		

**b**	**Df**	**SS**	**R^2**	**F**	**p-value**
**Model**	1	0.0432	0.1376	1.4354	**0.005**
**Residual**	9	0.2710	0.8625		
**Total**	10	0.3142	1.0000		

Table S8: Mantel test using genetic distances and overland and river distances for the Occidental phylogeographic unit, Douro river system and the Sabor watershed showing the correlation coefficient (r) and p-value for each test. Values with p < 0.01 are highlighted in bold. Missing values (-) are due to differences in the spatial context of the analyses. For the Occidental phylogeographic unit, river distances were not calculated because the samples are distributed across different river systems, making direct river-based distances biologically meaningless. To disentangle the relative contributions of overland and river distances for the Douro river system, partial Mantel tests were performed: (i) testing the effect of overland distance on genetic distance while controlling for river distance (Overland distances), and (ii) testing the effect of river distance on genetic distance while controlling for overland distance (River distances).

	**Simple Mantel test**				**Partial Mantel test**			
	**Overland distances**		**River distances**		**Overland distances**		**River distances**	
	**r**	**p-value**	**r**	**p-value**	**r**	**p-value**	**r**	**p-value**
**Occidental phylogeographic unit**	0.7884	**0.0001**	-	-	-	-	-	-
**Douro river system**	0.3403	0.0174	0.4206	**0.0026**	0.3403	0.0180	0.4160	**0.0020**
**Sabor watershed**	0.4461	0.0553	0.7039	**0.0001**	-	-	-	-

References

1. Escoda L, Castresana J. The genome of the Pyrenean desman and the effects of bottlenecks and inbreeding on the genomic landscape of an endangered species. Evol Appl. 2021;14:1898–913.
2. Querejeta M, González-Esteban J, Gómez A, Fernández-González A, Aymerich P, Gosálbez J, et al. Genomic diversity and geographical structure of the Pyrenean desman. Conserv Genet. 2016;17:1333-44.
3. Escoda L, Hawlitschek O, González-Esteban J, Castresana J. Methodological challenges in the genomic analysis of an endangered mammal population with low genetic diversity. Sci Rep. 2022;12:21390.
4. Escoda L, Gonzalez-Esteban J, Gomez A, Castresana J. Using relatedness networks to infer contemporary dispersal: Application to the endangered mammal *Galemys pyrenaicus*. Mol Ecol. 2017;26:3343-57.
