# Supplementary material for: Microgeographic genomic variation and connectivity in an endangered semiaquatic mammal
Source: BMC Ecol Evol. 2025 Oct 23;25:114. doi: 10.1186/s12862-025-02460-1 (PMC12548277; doi:10.1186/s12862-025-02460-1)
Supplement: Supplementary file 2 — Supplementary Material 2: Fig. S1: Diagram showing the flow of analyses for this manuscript. Fig. S2: Neighbour-joining tree based on the 115 samples and 110 SNPs using PHYLIP v3.6 with 100 bootstraps and the Russian desman (Desmana moschata) as outgroup. Bootstrap values from 100 replicates are displayed for nodes with support ≥ 50%. The five phylogeographic units are highlighted. Fig. S3: Bayesian Information Criterion (BIC) values for each number of clusters for the Discriminant Analysis of Principal Components analysis taking 6 principal components into account for the samples from the Occidental population, based on 7,604 SNPs. Fig. S4: Discriminant Analysis of Principal Components for the Occidental phylogeographic unit, with 6 principal components and based on 7,604 SNPs, showing 3 clusters. Fig. S5: STRUCTURE v2.3.4 analyses for the Occidental phylogeographic unit, based on 7,604 SNPs: (a) the rate of change in the likelihood of the data as K increases (Delta K) by Evanno et al. [1]; (b) the probability or likelihood of the data for each K (Prob(K)) based on the Bayesian clustering algorithm by Pritchard et al. [2]. Fig. S6: STRUCTURE v2.3.4 analyses for the Douro river system, based on 8,771 SNPs: (a) the rate of change in the likelihood of the data as K increases (Delta K) by Evanno et al. [1]; (b) the probability or likelihood of the data for each K (Prob(K)) based on the Bayesian clustering algorithm by Pritchard et al. [2]. Fig. S7: Scatterplots of genetic distance against geographic distances in the Douro river system. (a) Genetic distance versus overland distance, (b) genetic distance versus river distance, and (c) overland versus river distance. Each point represents a pair of individuals, coloured by watershed: Sabor (orange), Tua (blue), and across watersheds (green). Linear regression lines are shown for each category. These plots illustrate that river distances align more closely with genetic differentiation within watersheds, whereas overland di [file 12862_2025_2460_MOESM2_ESM.pdf]

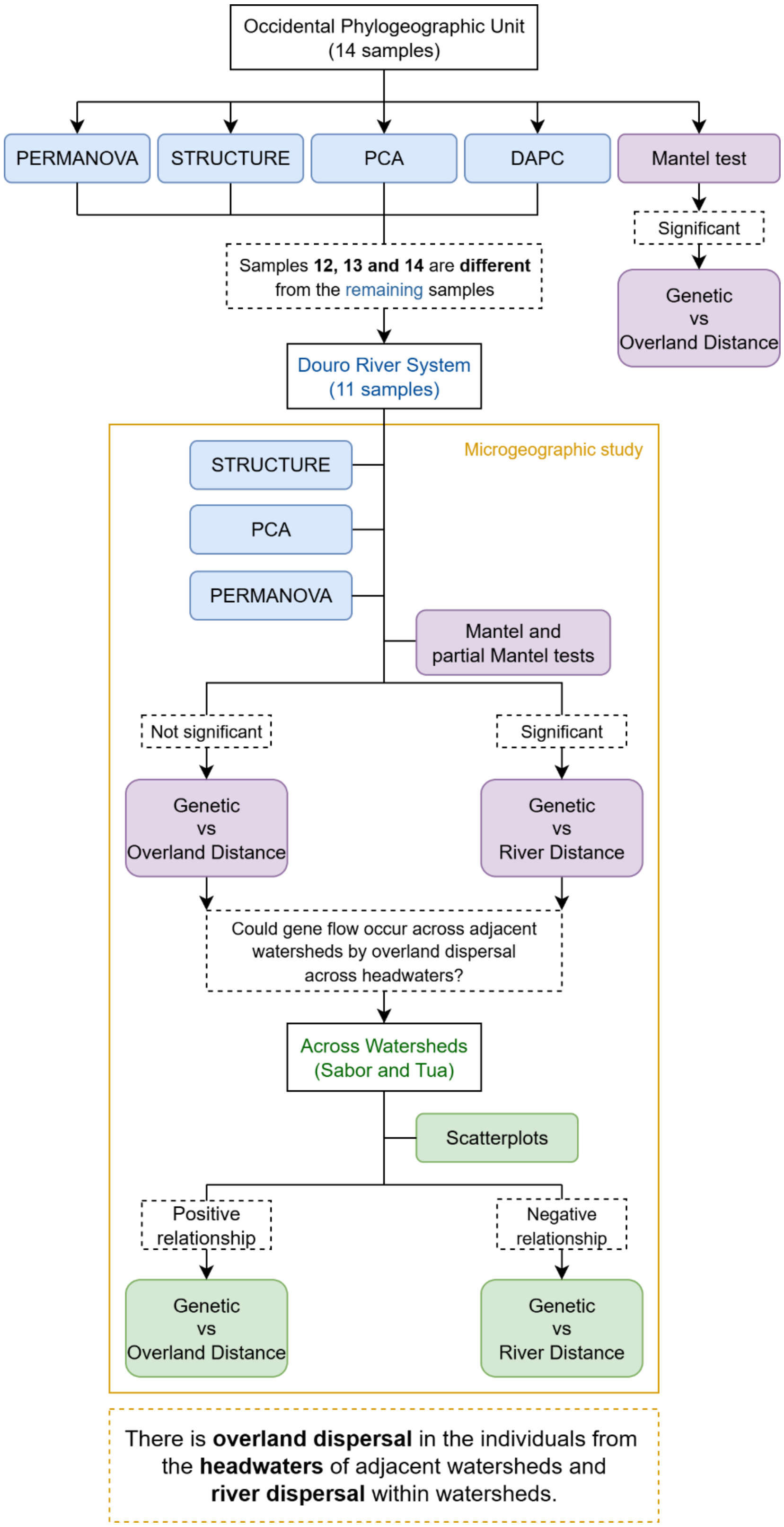

Fig. S1: Diagram showing the flow of analyses for this manuscript.

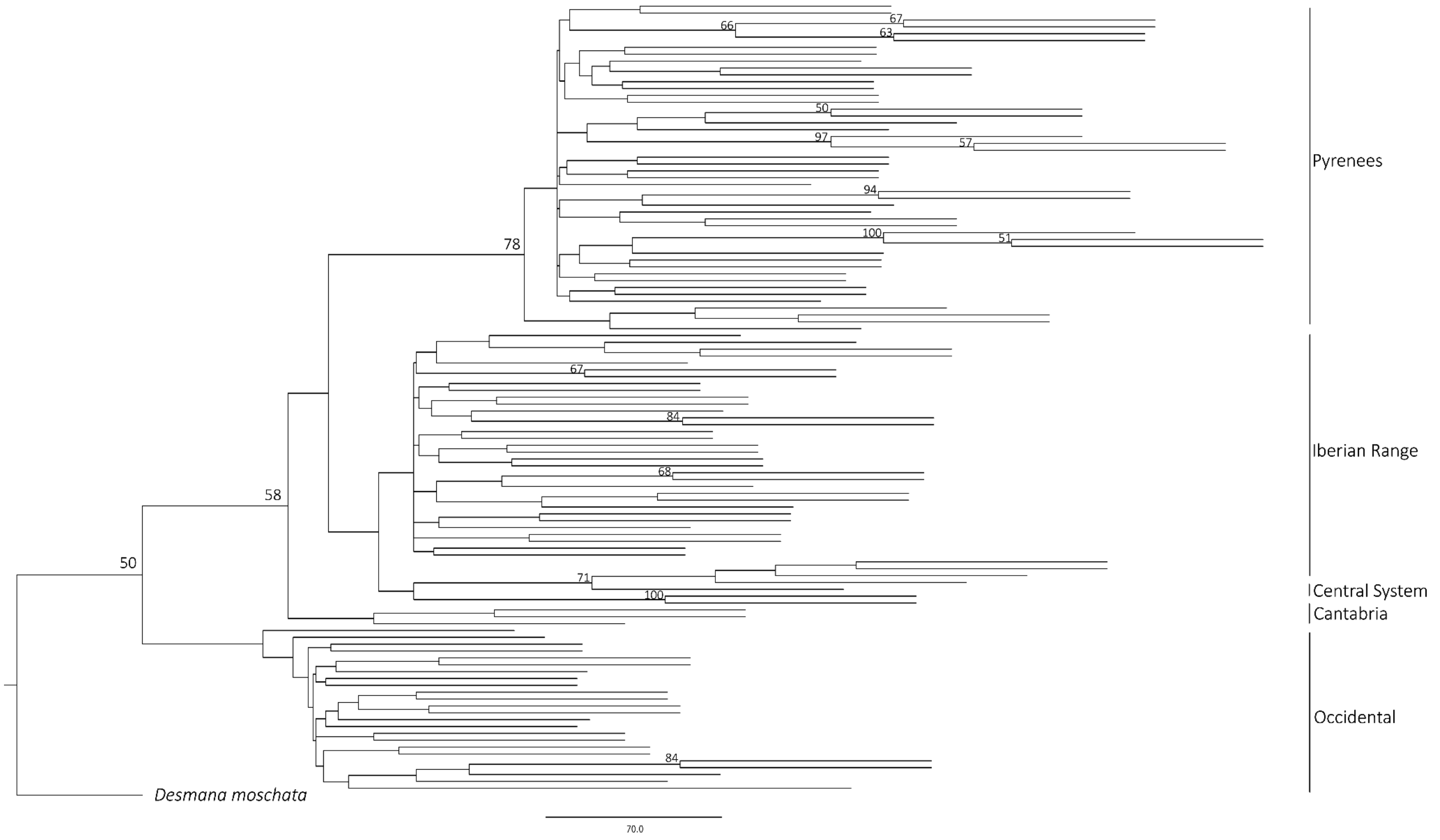

Fig. S2: Neighbour-joining tree based on the 115 samples and 110 SNPs using PHYLIP v3.6 with 100 bootstraps and the Russian desman (*Desmana moschata*) as outgroup. Bootstrap values from 100 replicates are displayed for nodes with support ≥ 50%. The five phylogeographic units are highlighted.

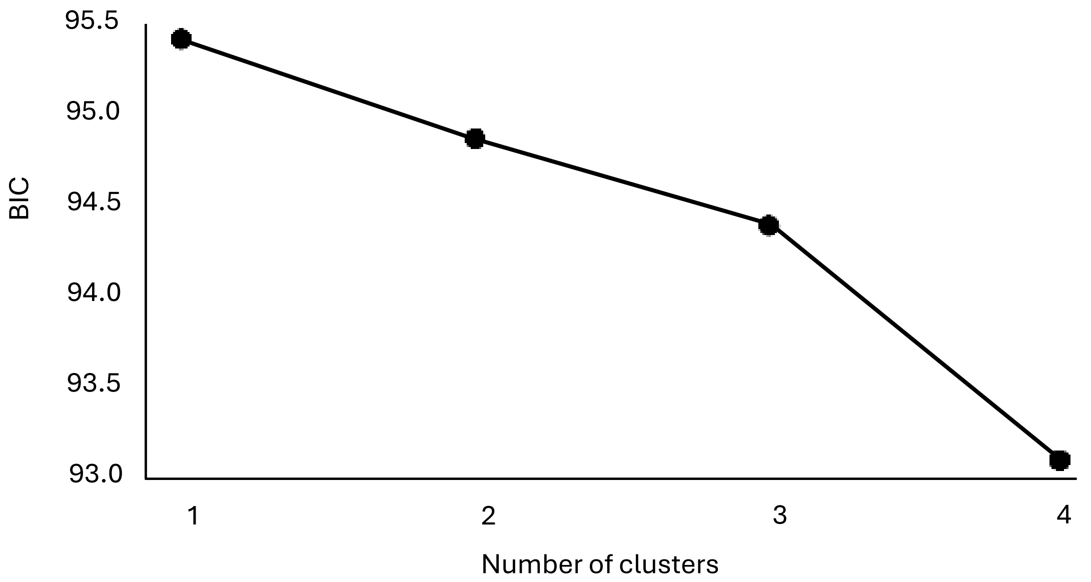

Fig. S3: Bayesian Information Criterion (BIC) values for each number of clusters for the Discriminant Analysis of Principal Components analysis taking 6 principal components into account for the samples from the Occidental population, based on 7,604 SNPs.

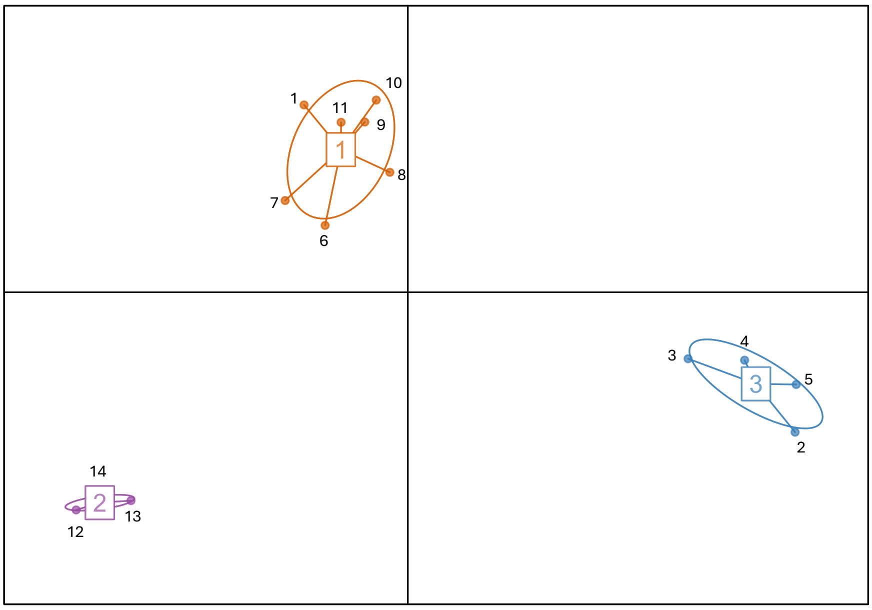

Fig. S4: Discriminant Analysis of Principal Components for the Occidental phylogeographic unit, with 6 principal components and based on 7,604 SNPs, showing 3 clusters.

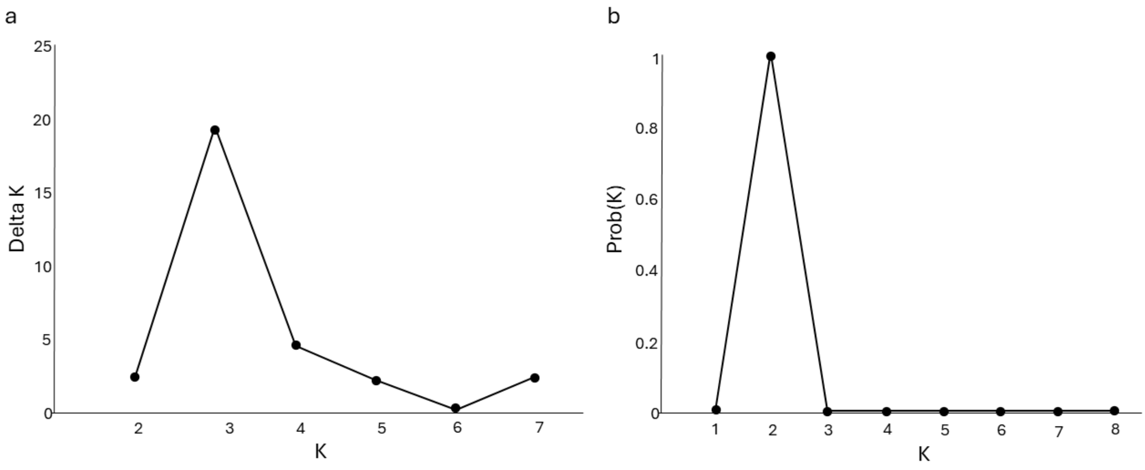

Fig. S5: STRUCTURE v2.3.4 analyses for the Occidental phylogeographic unit, based on 7,604 SNPs: **a.** the rate of change in the likelihood of the data as K increases (Delta K) by Evanno et al. [1]; **b**. the probability or likelihood of the data for each K (Prob(K)) based on the Bayesian clustering algorithm by Pritchard et al. [2].

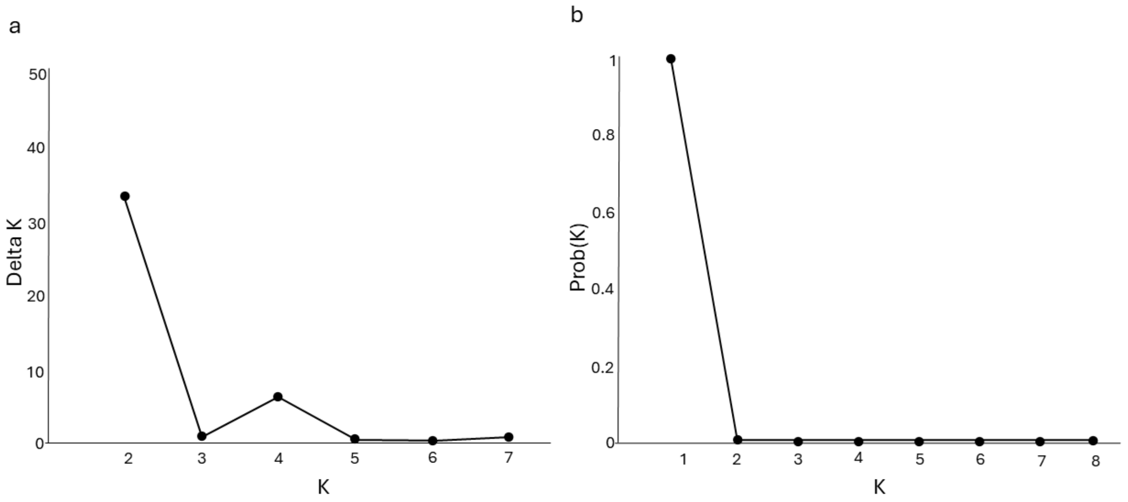

Fig. S6: STRUCTURE v2.3.4 analyses for the Douro river system, based on 8,771 SNPs: **a.** the rate of change in the likelihood of the data as K increases (Delta K) by Evanno et al. [1]; **b**. the probability or likelihood of the data for each K (Prob(K)) based on the Bayesian clustering algorithm by Pritchard et al. [2].

Fig. S7: Scatterplots of genetic distance against geographic distances in the Douro river system. (a) Genetic distance versus overland distance, (b) genetic distance versus river distance, and (c) overland versus river distance. Each point represents a pair of individuals, coloured by watershed: Sabor (orange), Tua (blue), and across watersheds (green). Linear regression lines are shown for each category. These plots illustrate that river distances align more closely with genetic differentiation within watersheds, whereas overland distances provide a better fit for between-watershed comparisons, consistent with both river-mediated and short-distance overland dispersal.

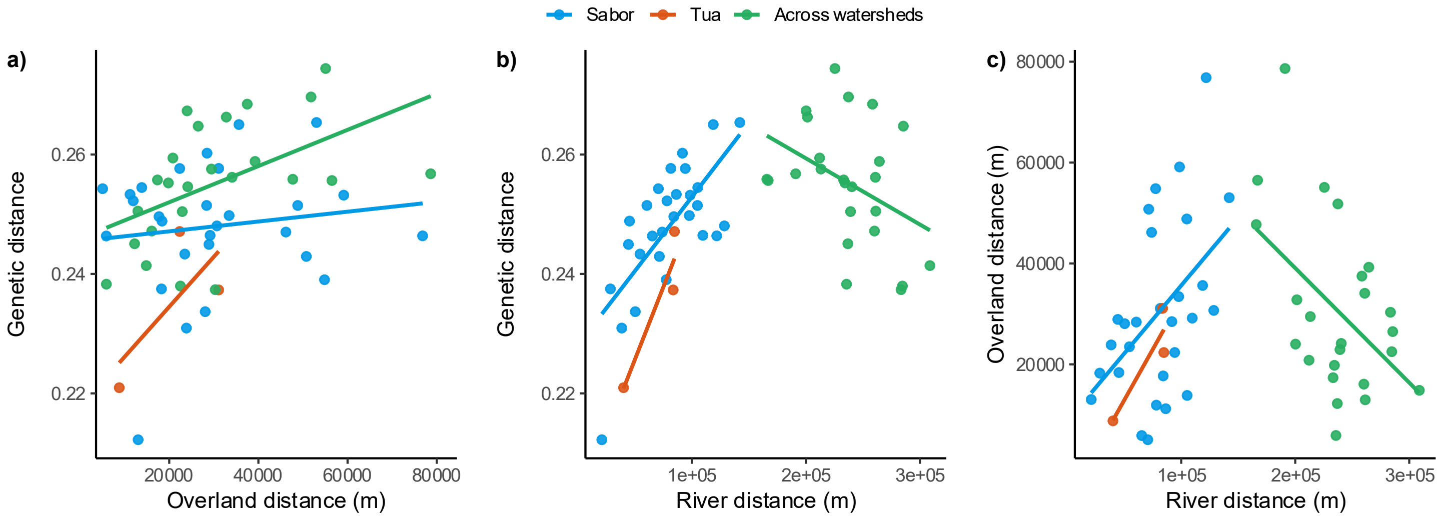

References

1. Evanno G, Regnaut S, Goudet J. Detecting the number of clusters of individuals using the software STRUCTURE: a simulation study. Mol Ecol. 2005;14:2611-20.

2. Pritchard JK, Stephens M, Donnelly P. Inference of population structure using multilocus genotype data. Genetics. 2000;155:945–59.
